# Intra- and extrapulmonary lipopolysaccharides-induced acute lung injury and pharmacotherapeutic response patterns in ventilated 7-day-old rabbits

**DOI:** 10.3389/ebm.2026.10788

**Published:** 2026-02-24

**Authors:** Guiyin Zhuang, Qiang Gu, Siyu Xie, Xiaojing Guo, Bo Sun

**Affiliations:** 1 Department of Pediatrics, The First Affiliated Hospital of Shihezi University, Shihezi, Xinjiang, China; 2 Department of Pediatrics, National Health Commission Laboratory of Neonatal Diseases, National Children’s Medical Center and Children’s Hospital of Fudan University, Shanghai, China

**Keywords:** ARDS, infection, inhaled nitric oxide, lipopolysaccharides, pulmonary surfactant

## Abstract

We explored pharmacotherapeutic response patterns of lipopolysaccharides (LPS)-induced pneumonia and sepsis as direct and indirect acute lung injury (ALI), and efficacy of a combined surfactant (S) and inhaled nitric oxide (iNO), simulating critical care, in rabbits of post-neonatal infancy. Anaesthetized 7-day-old healthy rabbits were injected intratracheally (IT) or intravenously (IV) with LPS (15–20–25 mg/kg, L) or saline as a control (C), and subjected to initial 2-hour mechanical ventilation (MV) with standardized tidal volume to induce ALI. They were then treated with S (200 mg/kg) and iNO (10 ppm, N), or not, thereby allocating to 6 groups (ITC, ITL, ITLSN, IVC, IVL, IVLSN) for another 8 h. Survival time/rate (ST), and variables as biomarkers in lung physiology, histopathology, biochemistry, and pathophysiology were measured. The survival was LPS-route, but not dosing, dependent. Compared to the IVL, ITL had relatively higher ST, lung injury score (LIS), lower intrapulmonary phospholipid pools, mRNA expressions in surfactant proteins (SPs) and pulmonary vascular endothelial cell injury (VEI)-related variables. ITLSN had higher phospholipid pools but no improvement in ST, lung mechanics, LIS or mRNA expression of SPs, proinflammatory mediators and VEI-related variables. IVLSN had improved lung mechanics, LIS, phospholipid pools, and SP-A mRNA expression, but worse ST, metabolic acidosis, higher interleukin mRNA expression in the lungs, liver and kidney, suspected as sepsis-associated multiorgan involvement. Using the infant rabbit LPS-ALI model, we characterized the survival as LPS-route dependent, the lung impairment and response pattern in surfactant and iNO treatment ineffectiveness/failure, as pharmacotherapeutic response patterns, with causal implication pertinent to the underlying pathophysiology of experimental pediatric ARDS.

## Impact statement

Pathophysiology and pharmacotherapeutic efficacies of ARDS requiring critical care in neonatal and post-neonatal infancy are unclear. In ventilated 7-day-old rabbits, direct (intratracheal) LPS-induced ALI was associated with severe pneumonia but moderate death risks whereas indirect (intravenous) LPS-ALI was with moderate lung injury but high deaths due to sepsis associated multiorgan impairment. Surfactant and inhaled NO failed to improve the survival, associated with highly provoked mRNA expression of proinflammatory mediators as multiorgan involved septic lung damage pattern. The pathophysiological and pharmacotherapeutic response patterns of LPS-route dependent worse outcome implicates causal relations underlying the mortality of experimental pediatric ARDS.

## Background

Acute respiratory distress syndrome (ARDS) as a severe and persistent hypoxemic respiratory failure (PHRF) is characterized by pulmonary infection, inflammation, high vascular-to-alveolar permeability and ventilation/perfusion mismatching. Its initial stage is acute lung injury (ALI) or mild-to-moderate ARDS. Current knowledge of etiology, pathogenesis and pathophysiology of ALI and PHRF is focused on direct and indirect assaults to the lungs, also known as pulmonary and extrapulmonary causes. These involve bacterial and viral pneumonia, lung abscess, contusion, airway obliteration, sepsis, acute pancreatitis, acid aspiration of gastric regurgitation, or embolism of bone fracture, etc.; and hemodynamic derangement as sepsis and septic shock with multiorgan system dysfunction/failure [1–4]. Its clinical definition has been modified several times, and response to oxygenation, high positive end-expiratory pressure (PEEP) and restricted tidal volume (V_T_) in mechanical ventilation (MV) are considered protective as mainstay [5].

There are differences in pathogenesis, pathophysiology, respiratory mechanics, histological and biochemical alterations for triggers attributable to pulmonary (ARDS_p_) and extrapulmonary ARDS (ARDS_exp_) [6, 7]. In ARDS_p_, the primary attack is due mainly to pathogen-associated response pattern in alveolar epithelial and capillary endothelial cells, causing wide spread activation of alveolar macrophages and the inflammatory response, confined mainly in the alveoli and small airway compartment [8, 9]. The damage to alveolar epithelial cells leads to decreased clearance of edema fluid from the alveolar space of injured blood-gas barrier, inactivation or depressed production of pulmonary surfactant (PS) by type II alveolar epithelial cells (ATII), with degenerative, suppurative and necrotic bronchiolitis and alveolitis, and ultimately remodeling with cystic pulmonary fibrosis. In contrast, ARDS_exp_ is caused by the release of inflammatory mediators from extrapulmonary assaults and organ tissue lesions, which primarily affect pulmonary capillary endothelial cells, then to ATs. The triggers cause massive production of inflammatory cells and mediators into the bloodstream leading to inflammatory cascade and aggregation of inflammatory cells, resulting in an increased permeability in blood-gas barrier, intrapulmonary edema, and microvascular tone dysregulation [8, 9]. Regarding the lung mechanics in ARDS_p_, dynamic compliance of respiratory system (Cdyn) is generally reduced even if high PEEP is applied whereas in ARDS_exp_ Cdyn would be more associated with the management of high permeability of blood-gas barrier and systemic hemodynamic derangement to assist in lung protective ventilation [10].

Postnatal maturation of lung structure and immune system likely influence the age-related response differences in lung and systemic infection susceptibility. This pertains to mechanisms of pulmonary inflammation, injury, reparation and remodeling in pediatric and infant patients to bacterial, viral and other pathogen induced ALI and PHRF, especially in late neonatal and postneonatal infancy [4, 11]. Moreover, it is unclear how the underlying mechanisms of pharmacotherapeutic action would be, with regard to ancillary respiratory medications in critical care for pediatric and neonatal patients at high death risk of ARDS. The consensus agreement on the definition of pediatric ARDS (pARDS), by the Pediatric Acute Lung Injury Consensus Conference (PALICC) clarified incidence, epidemiology, management, prognosis and outcome assessment [4, 5]. Surfactant therapy is recognized as an optimal treatment in lung protective ventilation strategies for neonatal respiratory distress syndrome (RDS) of prematurity [12]. However, in those from late neonatal to early post-neonatal infancy, there is insufficient evidence supporting the therapeutic efficacy of surfactant treatment for ALI and PHRF, even contradictory in interpretation of its efficacy [13]. Similarly, the existing evidence on the effectiveness of inhaled nitric oxide (iNO) in treatment of pARDS is also in debate due to transient improvement in oxygenation without reducing mortality but raised concerns on the extrapulmonary organ system safety [14]. Therefore, we speculate that patients diagnosed as pARDS may differ from adult ARDS, especially in the lung impairment and treatment response patterns with sensitive pathobiological biomarkers as phenotypes to predict outcome and prognosis [11, 14, 15].

In this study, we aimed to establish a high dose lipopolysaccharides (LPS)-induced ALI model, in rabbits at post-neonatal infancy, with two different administration routes, of etiological distinction, by intratracheal or intravenous injection of LPS with MV, to simulate critical care for neonatal and pediatric patients in early infancy. We first hypothesized that severity of ALI should be associated with LPS route and dosage to lead a survival rate or median survival time (ST_50_) in reasonable length (within 10 h) of V_T_-restricted MV [16]. This should enable characterizing the severity of septic ALI as in pARDS, with survival status as a key outcome linking the two different etiologies and associated damage response patterns. We then hypothesized that a combined PS and iNO regimen would exert better effects compared to MV alone [16], and explored their mechanisms of pharmacotherapeutic action. With unexpected outcome thereof, we managed to explain mechanisms of ineffectiveness/failure similar in the pARDS trials [17–19].

## Materials and methods

### Experimental ethics, protocol and animal management

The study protocol was approved by the Ethics Committee of the Children’s Hospital of Fudan University (No. 2023350). Healthy New Zealand White rabbits, 7-day-old in early infancy, were provided by Shanghai Songlian Experimental Animal Center. The rabbit pups’ age for developmental stage was estimated, by body weight (BW) gain, corresponding to human infant around 2-month of age, assuming an infant rabbit around 40–50 postnatal days at weaning with BW gain corresponding to approximately 1 year-old human infant. The animals care followed the institutional laboratory routine management, according to Chinese regulations for experimental animal care and welfare in the medical research and ethics requirement.

**FIGURE 1:**
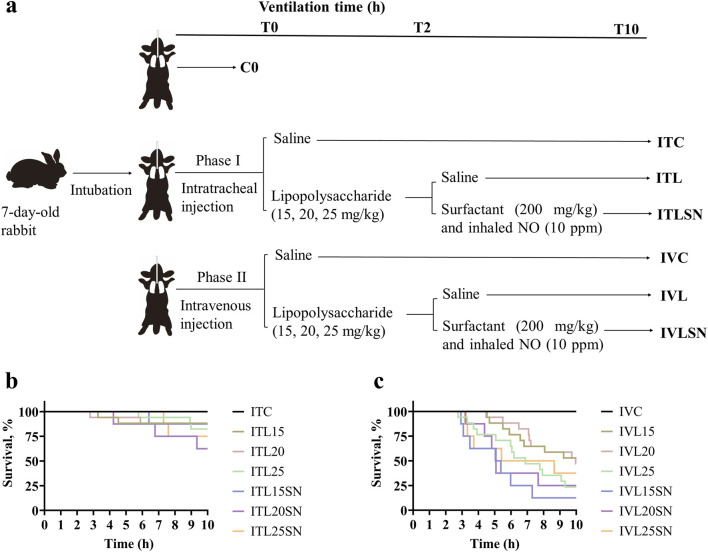
The study design and animal survival curves in Phase I and II experiments with different dosages and routes of lipopolysaccharides (LPS) administration. **(a)** Flow chart of the two phases of experiments with different LPS dosage and route of administration; **(b)** Survival curves of the animals receiving different dosage of LPS in the Phase I experiment (n = 8–17, ITC n = 12, ITL15/20/25 n = 17, ITLSN15/20/25 n = 8); **(c)** Survival curves of the animals receiving different dosages of LPS in the Phase II experiment (n = 8–17, IVC n = 12, IVL15/20/25 n = 17, IVLSN15/20/25 n = 8). Group definitions and abbreviations: C, control; IT, intratracheal; IV, intravenous; L, lipopolysaccharides (15, 20, 25 mg/kg body weight); N, inhaled nitric oxide (10 ppm); S, surfactant (200 mg/kg).

The pups were allocated to 7 groups: non-ventilated (C0), intratracheal saline (ITC), intratracheal LPS (ITL), intratracheal LPS + intratracheal PS + iNO (ITLSN), intravenous saline (IVC), intravenous LPS (IVL), and intravenous LPS + PS + iNO (IVLSN) (for pups’ average body weight and gender see Table 1). The experiments were conducted in two phases. In phase I, experiments were focused on direct, pulmonary ALI, simulating pneumonia-associated pARDS (pARDS_p_) as in the ITC, ITL and ITLSN groups. In phase II, experiments were focused on indirect, extrapulmonary-associated ALI, simulating sepsis-associated pARDS (pARDS_exp_), involving the IVC, IVL and IVLSN groups (Figure 1a).

**TABLE 1:** Basic information of experimental animals.

Group	N	Body weight (g)	Male n (%)
C0	12	118 ± 3.8	7 (58.3)
ITC	12	117 ± 5.8	3 (25.0)
ITL	51	114 ± 6.0	22 (43.1)
ITLSN	24	108 ± 4.4	5 (20.8)
IVC	12	115 ± 3.8	7 (58.3)
IVL	51	116 ± 5.5	25 (49.0)
IVLSN	24	108 ± 5.1	6 (25.0)

Group definitions and abbreviations: IT, intratracheal; IV, intravenous; C, control; L, lipopolysaccharides (15, 20, 25 mg/kg); S, pulmonary surfactant (200 mg/kg); N, inhaled nitric oxide (10 ppm); C0, non-ventilated control. Values are means ± standard deviation.

After weighing BW, pups were injected intraperitoneally with 3% sodium pentobarbital (1.5 mL/kg), and intratracheally intubated. In the phase I experiments, 2 mL/kg of saline was injected intratracheally in the ITC. *E. coli* (serotype 0111 B4, cat. No. L2630, Sigma-Aldrich, St. Louis, MO, USA)-derived LPS was dissolved in sterile saline to 7.5, 10 and 12.5 mg/mL, 2 mL/kg (corresponding to 15, 20 and 25 mg/kg, or 20 mg/kg in average, see below) was injected intratracheally into each pup lungs to induce ALI in the ITL and ITLSN groups. After LPS or saline injection, all the pups were connected to ventilator for MV. Two hours after the LPS injection and MV, pups in the ITLSN were intratracheally injected with 2.5 mL/kg of PS (200 mg/kg) and iNO (10 parts per million in volume, ppm), through inspiratory limb of the ventilator circuit and MV was resumed [20]. In the phase II experiment, each pup in the IVC received 2 mL/kg of saline injected through external cervical vein. For the pups in the IVL and IVLSN, each was injected intravenously with the same dose of LPS as used in the phase I. Two hours after LPS injection and MV, each pup in IVLSN received the same dose of PS and iNO as in ITLSN. After the administration of saline or LPS, the pups in both phases were subjected to the identical MV settings throughout. In each phase, several pups were immediately euthanized by intracranial injection of an overdose of 3% sodium pentobarbital to cause heart arrest, and served as a non-ventilated control group (C0). Thus, there were totally 186 rabbit pups enrolled, 12 each in the C0, IVC and ITC, 51 each in the IVL and ITL, and 24 each in the IVLSN and ITLSN groups.

### Mechanical ventilation, measurement of lung mechanics, and survival time

A multi-plethysmograph-ventilator system was used to enable a parallel MV, which consisted of specially designed 10 plexiglass boxes and a Servo ventilator (900C, Siemens-Elema, Solna, Sweden) to ventilate ten rabbits simultaneously [21]. The system also consisted of a pneumotachometer (RSS100-HR, Hans Rudolph Inc., Kansas City, KA), a pressure transducer (Shanghai Yangfan Electronic, Shanghai, China), and a bioelectric signal amplifier. Variables of lung mechanics were monitored and recorded by an automated physiologic monitoring system (PowerLab, ADInstruments Pty Ltd, New South Wales, Australia) with an electrocardiogram (ECG) module. Tidal volume (Vt), positive end-expiratory pressure (PEEP), and peak insufflation pressure (PIP) were measured through individual adjustment of PIP to achieve designated Vt in pups. Pressure-control mode was chosen with a fraction of inspired oxygen (FiO_2_) at 1.0, a respiratory rate of 40 beats/minute, an inspiratory to expiratory time ratio of 1:2, and Vt targeted at 4–6 mL/kg [22]. Dynamic compliance of respiratory system (Cdyn) was derived from Vt/(PIP-PEEP) corrected by BW, and expressed as mL/kg/cmH_2_O. During MV, 3% sodium pentobarbital was injected intraperitoneally as required to maintain analgesia while a stable spontaneous breathing was maintained. Throughout the MV, the rabbits were kept in the boxes on a 37 °C heating pad, and intraperitoneally injected per pup with 0.3 mL/kg mixed solution (10% dextrose and 5% sodium bicarbonate in the ratio of 6:4) every 1–2 h where necessary to counter balance acidosis and maintain basal metabolism. The time 0 was when the rabbits completed tracheal intubation and connected to the ventilator through the multi-plethysmograph system. Measurements of PIP, PEEP and Vt values were at initial 15–60 min, and every half hour thereafter, or as frequently as needed, until death or per protocol termination of the ventilation. The maximum time of MV was 10 h. During the ventilation, we carefully observed the face and body color of pups and their respiratory effort. If the animal appeared cyanotic or pale, heart rate was checked with electrocardiograph (ECG) (Supplementary Figure 1). At the termination of ventilation, pups were euthanized with an overdose of 3% sodium pentobarbital till heart arrest. Blood samples from the left heart ventricle was immediately withdrawn from left heart ventricle for blood gas analysis. The diagnostic criteria for ALI as early stage of ARDS were mainly characterized by deterioration of Cdyn by 30% from control level [23]. By histopathological features under light microscopy, there was a massive infiltration of inflammatory cells, widening of alveolar septa, alveolar atelectasis, and damage of alveolar septa, causing high vascular-to-alveolar permeability, as verified after initial 2-h of MV in additionally a few animals from both ITL and IVL groups [23, 24].

### Lung tissue sample processing

We used the same rabbits to undergo histopathological examination (right upper and lower lobes), and biochemical analysis (both lobes of left side), or quantitative PCR (qPCR) measurement of fresh tissue of right middle lobe. The lung accessory lobe was excised to determine the wet-to-dry weight ratio (W/D), which was used to assess the lung tissue fluid content. First, we ligated the right middle lobe to obtain lung tissue for qPCR or transmission electron microscopy. Next, we ligated the lung accessory lobe to obtain tissue for W/D ratio determination. Then, we ligated the right hilum and performed biochemical analysis on the left lung lobes. Finally, we ligated the left hilum and performed pressure fixation and sampling for histopathology (right upper and lower lobes). In some animals in each group, middle liver and cortical kidney tissues were collected for qPCR examination.

### Lung histopathology and morphometry

The lungs designated for histopathological analysis (n = 8–30 per group) were prepared for fixation by lung perfusion through a catheter placed in the pulmonary artery through right heart ventricle. Concurrently, a constant gas flow with pressure of 30 cmH_2_O was delivered via the intratracheal tube by the ventilator for 1 minute, then reduced to 10 cmH_2_O as the deflation pressure to maintain continuous alveolar expansion, approximating the functional residual volume. Lung tissue was perfused with a 4% paraformaldehyde solution for 30-min fixation, followed by further fixation in 4% paraformaldehyde, embedding in paraffin, sectioning, and staining with hematoxylin-eosin (H-E) to assess alveolar expansion (Vv), coefficient of variance of Vv [CV (Vv)], and lung injury score (LIS), with morphometry. Vv and CV (Vv) were counted in 30–40 fields using a light microscope (magnification ×200) and an IMS image analysis system (Leica, Wetzlar, Germany). The LIS was determined based on the severity of four items: edema, hemorrhage, inflammatory cell infiltration, and alveolar injury. The scale assigned a score of 0 for each injury item found areas less than 1%, 1 for 1%–25%, 2 for 25%–50%, 3 for 50%–75%, and 4 for >75% within the field. The LIS_total_ was calculated individually by summing up the four injury item scores [20].

### Ultrastructural morphology of the lungs

The lung tissue from the right middle lobe was freshly excised and trimmed to 1 mm^3^. It was then fixed in glutaraldehyde for 24 h at 4 °C and osmium, followed by dehydration, embedding, sectioning, staining, and examination with a transmission electron microscope (FEI Tecnai G2 Spirit TWIN, ThermoFisher Scientific, Boston, MA, USA).

### Biochemical analysis of phospholipids and proteins

For the left lung lobes subjected to biochemical analysis (n = 8–30 per group), by clamping right hilar, bronchoalveolar lavage (BAL) was performed with cold saline each time till full lung lobe expansion, followed by gentle withdrawal. Collected BAL fluid (BALF) was recorded. This procedure was repeated three times which yielded a total volume as recovered BALF, and subjected to measurement of total phospholipids (TPL) and disaturated phosphatidylcholine (DSPC) [25, 26]. Total proteins (TP) in BALF were determined using BCA kit (Thermo Fisher Scientific, Waltham, MA). When performing whole-lung lavage, the volume of normal saline instilled was 30 mL/kg; for a single lung, the volume was 15 mL/kg. In our experiment, only the left lung was lavaged. Therefore, to calculate whole-lung TPL or DSPC content, all the measurements were corrected for both lungs (as divided by 0.45), and its amount is expressed as mg/kg BW [27].

### Wet to dry weight ratio (W/D) determination

For W/D determination, the lung accessory lobe was weighed within 10 min of collection: surface moisture was gently removed with filter paper, and the wet weight (W) was recorded to 0.1 mg on a high-precision balance. The lung accessory lobe was then dried in a forced-air oven at 60 °C for ≥24 h until consecutive weighings were constant. Its final value was taken as dry weight (D, to 0.1 mg) and used to calculate W/D.

### Quantitative PCR of mRNAs

For the lung, liver, kidney tissue samples from the rabbits allocated to qPCR analysis (n = 5–40 per group), mRNA in fresh organ (lung, liver, kidney) tissue samples were extracted using RNAex Pro Reagent (Accurate Biotechnology CO., LTD, Changsha, China) following the manufacturer’s instructions. Total RNA was reverse-transcribed into single-stranded cDNA using Evo M-MLV PrimeScript RT reagent kit, following the manufacturer’s instructions. Total RNA levels were measured with an SYBR green detection system using a Roche LightCycler 480 II system (Roche, Basel, Switzerland). The target genes were categorized as follows: surfactant proteins (SP)-A, SP-B, SP-C, nuclear transcript factor kappa B (NF-κB), tumor necrosis factor (TNF)-α, interleukin (IL)-1β, IL-6, IL-8, tyrosine kinase receptor (Tie)-2, angiopoietin (Ang)-1 and Ang-2. The mRNA levels of each gene were standardized against β-actin expression, employing 2^−△△Ct^ formula to determine average fold change in reference to the control (C0) group. Primer sequences are provided in the Supplementary Table 1.

### Statistics and data analysis

Statistical analyses were performed using SPSS version 26.0 (IBM, Armonk, NY), and figures were produced by GraphPad Prism 9.0 (GraphPad Software, La Jolla, CA). For survival analysis, the Kaplan-Meier survival curve was applied, complemented by log-rank test. Median survival time (ST_50_) is expressed as the survival length of 50th percentile in a group number, denoting as median survival time, and corresponding interquartile range (IQR as 75th and 25th percentiles), in minutes. Quantitative data were presented as means ± standard deviation (SD). Proportional data, including survival rate, were presented as number (n) and percentage (%). For quantitative data, One-way ANOVA F test with Bonferroni *post hoc* test, or Kruskal-Wallis H test with Wilcoxon-Mann-Whitney U test, was utilized, depending on the continuity, distribution characteristics, variance homogeneity of data, and repeated measures. *P* value <0.05 was considered statistically significant for the difference.

## Results

In the initial experiment (ITL and IVL) (Supplementary Figures 2, 3; Supplementary Tables 2, 3), we found that the LPS induced ALI severity was not dose-dependent, neither were the survival, phospholipid content in BALF, or the mRNA expression of pro-inflammatory cytokines and mediators in lung tissue. Therefore, to facilitate data analysis and presentation, the groups of different doses of LPS were combined, hence the number of animals, and designated as 20 mg/kg of LPS in average in the ITL, ITLSN, IVL and IVLSN groups, respectively, in the main results for easy comprehension (Figures 1b,c). Average BW was around 115 g, which was approximately doubled from that at term birth (31 days of gestation, with birth weight of 55–65 g in average), and male-to-female was 4:6. Values of W/D were significantly lower in the IVL than in the ITL (Table 2).

**TABLE 2:** General status of the experimental animals in various groups.

Group	N	Survival (%)	pH	PCO_2_ (mmHg)	Lactate (mmol/L)	W/D
C0	12	​	7.20 ± 0.12	74.3 ± 15.9	5.1 ± 3.5	5.06 ± 0.42
ITC	12	12 (100)	7.29 ± 0.18	65.2 ± 11.1	3.6 ± 2.7	5.33 ± 0.52
ITL	51	44 (86.3)	7.25 ± 0.18	63.8 ± 17.8	4.4 ± 5.0	5.48 ± 0.41
ITLSN	24	18 (75)	7.28 ± 0.14	56.9 ± 19.6	6.8 ± 6.2	5.51 ± 0.43
IVC	12	12 (100)	7.30 ± 0.06	62.4 ± 16.7	4.1 ± 3.3	5.51 ± 0.30
IVL	51	21 (41.2)	7.19 ± 0.15	43.5 ± 21.7^!###^	12.5 ± 5.8^!!!###^	5.25 ± 0.32^#^
IVLSN	24	6 (25)	7.20 ± 0.20	37.0 ± 21.1	13.0 ± 6.0	5.26 ± 0.43

Group definitions and abbreviations: IT, intratracheal; IV, intravenous; C, control; L, lipopolysaccharides (15, 20, 25 mg/kg); S, pulmonary surfactant (200 mg/kg); N, inhaled nitric oxide (10 ppm); C0, non-ventilated control. Values are means ± standard deviation. W/D, wet/dry lung weight ratio. ^#^ *P* < 0.05, ^###^ *P* < 0.001 vs. ITL, ^!^ *P* < 0.05, ^!!!^ *P* < 0.001 vs. IVC.

### Phase I experiment: IT-LPS

#### Survival status and blood gas analysis

**FIGURE 2:**
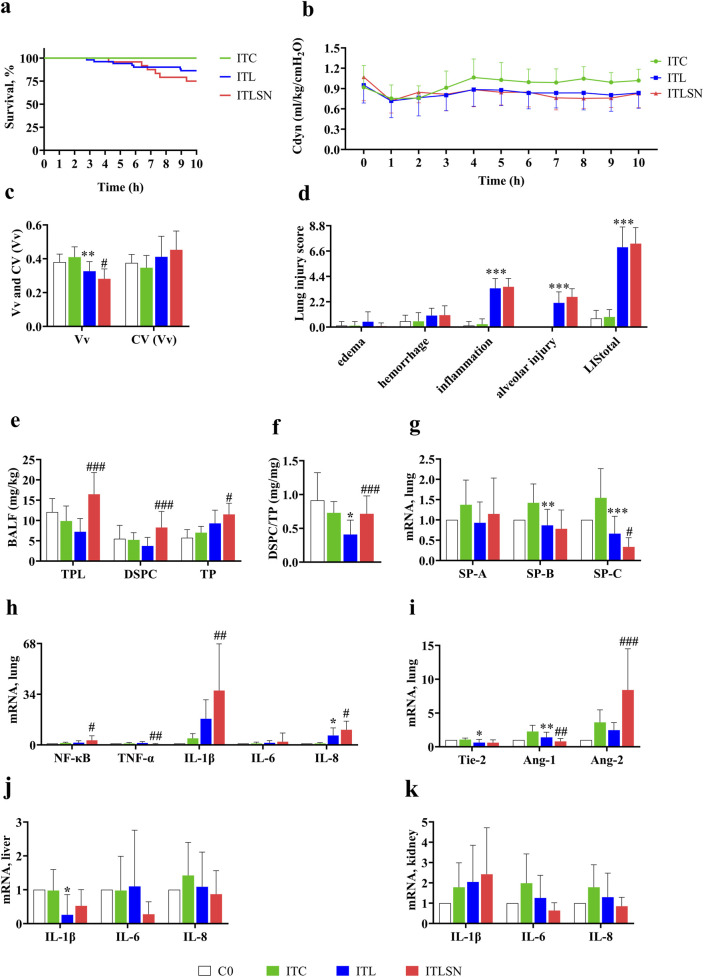
Major results of 7-day old rabbits in Phase I experiment. **(a)** Kaplan-Meier survival curves (ITC n = 12, ITL n = 51, ITLSN n = 24), ITC, saline control; ITL, combined intratracheal LPS 15–25 mg/kg; ITLSN, ITL with surfactant and iNO treatment; **(b)** Trend of dynamic compliance of respiratory system (Cdyn) during ventilation (for details with sample size, see Supplementary Table 4) (ITC n = 12, ITL n = 51, ITLSN n = 24). **(c)** Alveolar expansion (Vv) and variation of alveolar aeration (CV(Vv)) (C0 n = 8, ITC n = 8, ITL n = 30, ITLSN n = 24); **(d)** Lung injury score (LIS) (C0 n = 8, ITC n = 8, ITL n = 30, ITLSN n = 24); **(e)** Total phospholipids (TPL), disaturated phosphatidylcholine (DSPC) and total proteins (TP) in bronchoalveolar lavage fluid (BALF) (C0 n = 8, ITC n = 7, ITL n = 30, ITLSN n = 24); **(f)** DSPC/TP ratio; **(g)** mRNA expression of surfactant proteins (SP) in lung tissue (C0 n = 8, ITC n = 8, ITL n = 30, ITLSN n = 24); **(h)** mRNA expression of pro-inflammatory mediators in lung tissue (C0 n = 8, ITC n = 8, ITL n = 30, ITLSN n = 24); NF-κB, nuclear transcription factor-κB; TNF-α, tumor necrosis factor-α; IL, interleukin; **(i)** mRNA expression of the injury marker of endothelial cells in lung tissue: Tie-2, tyrosine kinase receptor-2; Ang, angiopoietin; **(j)** mRNA expression of pro-inflammatory mediators in liver tissue (C0 n = 5, ITC n = 6, ITL n = 40, ITLSN n = 19); **(k)** mRNA expression of pro-inflammatory mediators in kidney tissue (C0 n = 5, ITC n = 6, ITL n = 40, ITLSN n = 19). For group definitions see Figure 1 legends. Values are mean ± standard deviation. *P < 0.05, **P < 0.01, ***P < 0.001 vs. ITC, ^#^ *P* < 0.05, ^##^ *P* < 0.01, ^###^ *P* < 0.001 vs. ITL (Due to the very small mean ± standard deviation of the control group, the value levels are almost imperceptible in the figure d and h).

There were no deaths in the ITC during the 10-h MV period. The survival rates in ITL and ITLSN groups were low, however both being above 50% (Figures 1b, 2a; Table 2), and not the LPS-dose dependent. No significant differences in pH, PCO_2_ or lactate levels were observed among the ITC, ITL and ITLSN groups (Table 2). By subgroup analysis for the early death survived less than 10 h, those from ITL and ITLSN were more acidotic and had higher lactate in the blood (Table 3), denoting metabolic acidosis.

**TABLE 3:** General status of the experimental animals. Stratified analysis of early death and 10-h survivors on blood gas and W/D.

Group	Survival h (n)	pH	PCO_2_ (mmHg)	Lactate (mmol/L)	W/D
ITL	<10 (6)	7.17 ± 0.18	45.7 ± 23.0^a^	14.1 ± 6.4^aaa^	5.31 ± 0.45
ITL	10 (39)	7.26 ± 0.18	66.5 ± 15.4	3.0 ± 2.6	5.50 ± 0.41
ITLSN	<10 (6)	7.13 ± 0.20^b^	43.8 ± 26.7	15.2 ± 4.2^bbb^	5.40 ± 0.49
ITLSN	10 (17)	7.34 ± 0.04	61.5 ± 14.8	3.8 ± 3.3	5.55 ± 0.42
IVL	<10 (26)	7.21 ± 0.15	29.8 ± 13.9^ccc^	16.6 ± 3.0^ccc^	5.21 ± 0.32
IVL	10 (22)	7.17 ± 0.16	59.6 ± 18.0	7.6 ± 4.4	5.30 ± 0.33
IVLSN	<10 (18)	7.18 ± 0.20	28.9 ± 13.2^ddd^	15.1 ± 4.4^ddd^	5.17 ± 0.30
IVLSN	10 (6)	7.25 ± 0.21	61.6 ± 22.3	6.7 ± 5.9	5.55 ± 0.66

Group definitions and abbreviations: IT, intratracheal; IV, intravenous; C, control; L, lipopolysaccharides (15, 20, 25 mg/kg); S, pulmonary surfactant (200 mg/kg); N, inhaled nitric oxide (10 ppm); C0, non-ventilated control. Values are means ± standard deviation. W/D, wet/dry lung weight ratio. ^a^ *P* < 0.05, ^aaa^ *P* < 0.001 vs. ITL (10 h), ^b^ *P* < 0.05, ^bbb^ *P* < 0.001 vs. ITLSN (10 h), ^ccc^ *P* < 0.001 vs. IVL (10 h), ^ddd^ *P* < 0.001 vs. IVLSN (10 h).

#### Measurement of lung mechanics

During the MV period, Cdyn values were lower in ITL and ITLSN groups than in the ITC throughout. At the end, values of Cdyn of both groups were still 20% lower than that of the ITC group (*P* < 0.05, Figure 2b; Supplementary Table 4). Representative data of Cdyn are presented in Supplementary Table 4.

#### Lung histopathology and morphometry

The lung histopathology and morphometry of each group in the phase I are shown in Figures 2c,d, 4 (see below). The lungs from ITL and ITLSN groups demonstrated pronounced alveolar destruction alternatively with moderate alveolar septa thickening, and wide spread alveolar inflammatory cell infiltration, epithelial damage, along with overt infill of both alveoli and small airways, by exudative cells and fluid, supposedly from capillary lumen. Compared with the ITC, the ITL group had 20% lower Vv (*P* < 0.01) and 17% higher CV (Vv) values, whereas that in the ITLSN were even worse, both groups showing a significantly higher grade of inflammation, alveolar injury, and LIS_total_ values (all *P* < 0.001).

#### Ultrastructural observation of the lungs

With transmission electron microscopy observation, in the normal saline group, there were lamellar bodies (LBs) in clean and well aerated alveolar spaces (Figure 5a) and type I and II alveolar epithelial cells (ATI, ATII, respectively) with abundant capillaries. Tubular myelin is readily discernible at high magnification. In contrast, the alveolar spaces of the LPS-treated group were filled with many scattered cells and loosened ATI, ATII with fewer LB, and many interstitial cells with intensified lysosomes in cytoplasm, denoting inflammatory response to LPS (Figures 5b–e). In the alveolar space of the ITLSN, among scattered LB, there were ring-shaped membrane layers, much exceeding the adjacent epithelial cell size, likely exogenous surfactant phospholipids (Figure 5f). Tension expanded alveolar septa with surface fluid layer containing cell debris and LB alternative with infilled polymorphonucleated cells are present in alveolar space.

#### Biochemical analysis of phospholipids and proteins in BALF and lung homogenate (LH)

The TPL and DSPC levels in BALF were lower, and TP higher, in the ITL than in the ITC. The DSPC/TP in ITL was significantly lower. In the ITLSN, TPL and DSPC levels were more than 100% higher than the ITL (all *P* < 0.001), with 24% increment in TP, and 76% increment in DSPC/TP (ITL: TPL 7.24 ± 3.26, DSPC 3.73 ± 2.11, TP 9.30 ± 3.25, DSPC/TP 0.41 ± 0.21; ITLSN: TPL 16.5 ± 5.34, DSPC 8.28 ± 3.97, TP 11.5 ± 2.75 and DSPC/TP 0.72 ± 0.26) (Figures 2e,f; Table 4). DSPC/TPL was around 40–50% with no significant difference among the three IT-groups. In the total lung phospholipid pool (BALF + LH) of the C0, ITC and ITL groups, TPL were around 140–180 mg/kg, DSPC 35–50 mg/kg, and DSPC/TPL 25–29%, in average (Table 4). For TPL and DSPC in the ITLSN, there were 233 and 70 mg/kg in the total pools, which derives net increments by approximately 72 and 28 mg/kg, or 45% and 66%, in average over the TPL and DSPC of the ITC and ITL groups, respectively. Notably, there were ratios of 13 and 7 between LH and BALF (i.e. 216 vs. 16.5 and 62 vs. 8.3 mg/kg, Table 4) for TPL or DSPC, respectively, in the ITLSN.

**TABLE 4:** Analysis of lung phospholipid pools.

Group	N	TPL_BALF_ (mg/kg)	DSPC_BALF_ (mg/kg)	DSPC/TPL_BALF_ (%)	TP_BALF_ (mg/kg)	DSPC/TP_BALF_ (mg/mg)
C0	8	12.1 ± 3.34	5.50 ± 3.30	42.6 ± 18.3	5.74 ± 2.03	0.91 ± 0.41
ITC	7	9.89 ± 3.69	5.23 ± 1.77	55.2 ± 20.9	7.00 ± 1.56	0.73 ± 0.17
ITL	30	7.24 ± 3.26	3.73 ± 2.11	49.9 ± 19.9	9.30 ± 3.25	0.41 ± 0.21*
ITLSN	24	16.5 ± 5.34^###^	8.28 ± 3.97^###^	50.3 ± 13.7	11.5 ± 2.75^#^	0.72 ± 0.26^###^
IVC	8	8.48 ± 2.97	4.32 ± 2.24	49.1 ± 17.5	6.51 ± 1.89	0.65 ± 0.26
IVL	30	8.79 ± 2.30	4.26 ± 1.89	48.1 ± 16.4	6.15 ± 2.03^###^	0.69 ± 0.21^###^
IVLSN	24	32.2 ± 12.6^∧∧∧^	15.1 ± 5.96^∧∧∧^	47.0 ± 5.90	10.5 ± 4.20^∧∧∧^	1.51 ± 0.51^∧∧∧^

Group	N	TPL_LH_ mg/kg	DSPC_LH_ mg/kg	DSPC/TPL_LH_ %	TPL_BALF+LH_ mg/kg	DSPC_BALF+LH_ mg/kg	DSPC/TPL_BALF+LH_ %
C0	8	146 ± 24.2	37.1 ± 15.2	25.8 ± 9.70	158 ± 26.5	42.6 ± 13.4	27.3 ± 7.64
ITC	7	167 ± 46.0	44.1 ± 18.5	26.8 ± 9.78	177 ± 48.0	49.3 ± 18.8	28.2 ± 9.38
ITL	30	138 ± 45.4	31.9 ± 15.3	23.1 ± 8.32	145 ± 45.1	35.6 ± 15.2	24.7 ± 7.78
ITLSN	24	216 ± 62.7^###^	62.0 ± 33.5^###^	28.7 ± 11.9	233 ± 64.9^###^	70.3 ± 36.5^###^	30.0 + 11.5
IVC	8	123 ± 35.9	29.0 ± 9.61	25.1 ± 10.1	131 ± 34.0	33.3 ± 9.43	26.9 ± 10.5
IVL	30	152 ± 47.6	35.0 ± 15.8	23.7 ± 10.4	160 ± 48.1	39.3 ± 16.2	25.1 ± 9.61
IVLSN	24	240 ± 71.5^∧∧∧^	71.5 ± 36.3^∧∧∧^	28.6 ± 9.00	272 ± 68.3^∧∧∧^	86.6 ± 36.8^∧∧∧^	31.1 ± 7.75

Values are means ± standard deviation. TPL, Total phospholipid; DSPC, Disaturated phosphatidylcholine. TP, Total proteins. BALF, Bronchoalveolar lavage fluid; LH, Lung homogenate. *P < 0.05 vs. ITC, ^#^ *P* < 0.05, ^###^ *P* < 0.001 vs. ITL, ^∧∧∧^ *P* < 0.001 vs. IVL. For group definitions see Table 1 legends.

#### Measurement of mRNA expression in the lung tissue

The IL-1β mRNA expression was increased in ITL, and even higher in ITLSN, whereas that for IL-8 and Ang-2 in both groups had similar but moderately enhanced expression. That for SP-A, -B, -C were mildly enhanced in the ITC but significantly depressed in SP-C for ITLSN (Figures 2g–i).

#### Comparison of mRNA expression of pro-inflammatory cytokines in liver and kidney tissues

In general, the magnitudes of mRNA expression of IL-1β, IL-6 and IL-8 in the liver tissue of ITC, ITL and ITLSN groups were not enhanced or lower (Figure 2j). And for kidney, in the ITLSN there was a modest increment in IL-1β and moderate decrement in IL-6 and IL-8 expression (Figure 2k).

### Phase II experiment: IV-LPS

#### Survival status and blood gas analysis

**FIGURE 3:**
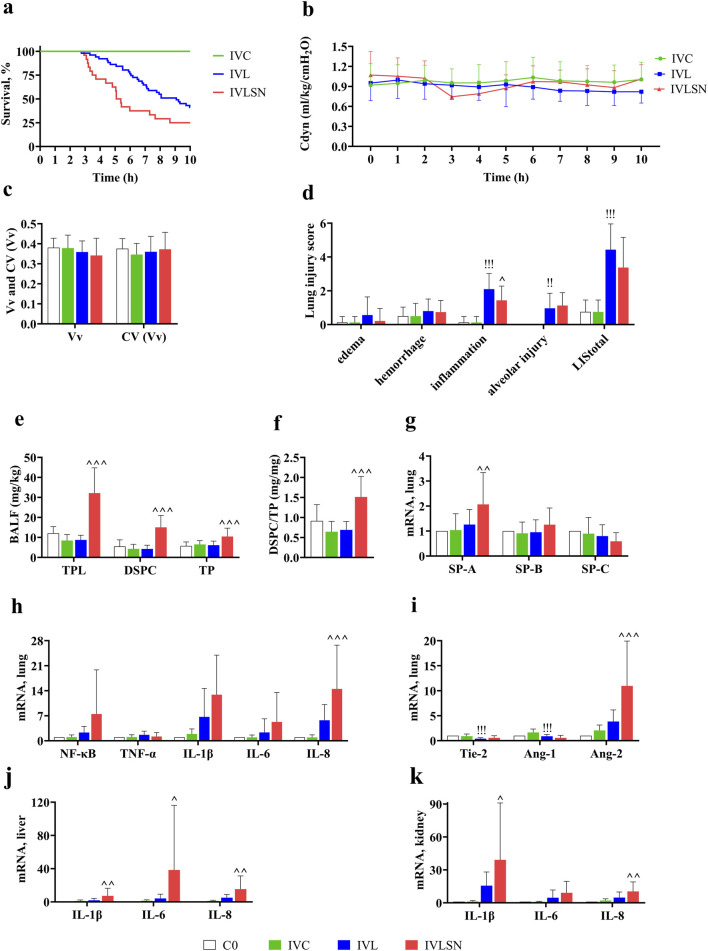
Major results of 7-day old rabbits in Phase II experiment. **(a)** Kaplan-Meier survival curves (IVC n = 12, IVL n = 51, IVLSN n = 24); IVC, saline control; IVL, combined intravenous LPS 15–25 mg/kg; IVLSN, IVL with surfactant and iNO treatment; **(b)** Trend of Cdyn during ventilation (for details with sample size, see Supplementary Table 4) (IVC n = 12, IVL n = 51, IVLSN n = 24); **(c)** Vv and CV(Vv) (C0 n = 8, IVC n = 8, IVL n = 30, IVLSN n = 24); **(d)** LIS (C0 n = 8, IVC n = 8, IVL n = 30, IVLSN n = 24); **(e)** TPL, DSPC and TP in BALF (C0 n = 8, IVC n = 8, IVL n = 30, IVLSN n = 24); **(f)** DSPC/TP ratio; **(g)** mRNA expression of SP (C0 n = 8, IVC n = 8, IVL n = 30, IVLSN n = 24); **(h)** mRNA expression of pro-inflammatory mediators in lung tissue (NF-κB, TNF-α, IL) (C0 n = 8, IVC n = 8, IVL n = 30, IVLSN n = 24); **(i)** mRNA expression of the injury markers of endothelial cells in lung tissue (Tie-2, Ang) (C0 n = 8, IVC n = 8, IVL n = 30, IVLSN n = 24); **(j)** mRNA expression of pro-inflammatory mediators in liver tissue (C0 n = 5, IVC n = 5, IVL n = 36, IVLSN n = 24); **(k)** mRNA expression of pro-inflammatory mediators in kidney tissue (C0 n = 5, IVC n = 5, IVL n = 36, IVLSN n = 24). For group definitions see Figure 1 legends; and for abbreviations see Figures 1, 2. Legends. Values are mean ± standard deviation. ^!!^ *P* < 0.01, ^!!!^ *P* < 0.001 vs. IVC, ^∧^ *P* < 0.05, ^∧∧^ *P* < 0.01, ^∧∧∧^ *P* < 0.001 vs. IVL. (Due to the very small mean ± standard deviation of the control group, the value levels are almost imperceptible in the figure **d**, **j** and **k**).

Like that in the phase I, there were no death in the IVC whereas the survival rates in the IVL and IVLSN groups were significantly decreased and below 50% (*P* < 0.01) (Figure 3a). The ST_50_ of the IVL was around 545 min, along with a survival rate of 41.2% at 10 h. There were further shortened ST_50_ at 314 min and an even lower survival rate of 25% in the IVLSN (Figure 3a) in contrast to ITLSN (Figure 2a; Table 2). By subgroup analysis for the early deaths (Table 3), those survived less than 10 h from the IVL and IVLSN groups had lower average PCO_2_ (30 mmHg) and high lactate (16–17 mmol/L) in contrast to those survived 10 h (60 mmHg, 7 mmol/L), respectively.

#### Measurement of lung mechanics

During the whole 10-h MV period, Cdyn in IVC was around 1.0 mL/kg/H_2_O with no deterioration. In IVL in the initial 5 h, Cdyn was very close to that of IVC, and in the late 5 h, it fell by 10–15% and ended at 0.82 in average. That for IVLSN an upward trend was observed during the late 5-h around 1.0, close to that of the IVC (Figure 3b; Table 4). The IVL and IVLSN had the lowest ST_50_, survival rate, pH and the highest lactate of the blood sample at termination of MV (Table 2).

#### Lung histopathology and morphometry

**FIGURE 4:**
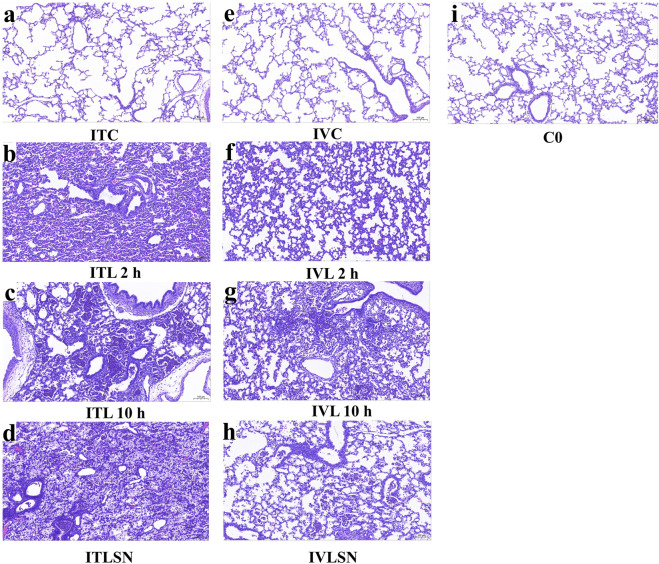
Representative photomicrographs of the lungs at different ventilation time. **(a)** ITC; **(b)** ITL 2 h; **(c)** ITL 10 h; **(d)** ITLSN; **(e)** IVC; **(f)** IVL 2 h; **(g)** IVL 10 h; **(h)** IVLSN; **(i)** C0. Scale bar = 100 μm. For group definitions see Figure 1 legends.

Figure 4 contains representative photomicrographs of the Phase I and II groups. No significant damage alterations were observed in the lungs of ITC groups. Both IVL and IVLSN groups exhibited moderate to severe lung pathologic changes compared with those in the ITL, as more aerated alveoli are seen, as measured by V_V_ and LIS in both IVL and IVLSN groups compared to that of ITL and ITLSN groups (Supplementary Figures 4a,b). Compared with the IVC, IVL did not show significant differences in Vv, CV (Vv), edema or hemorrhage scores, but had significant increases in inflammation, alveolar injury scores, and LIS_total_. No significant differences in Vv and CV (Vv) were observed between the IVL and IVLSN groups (Figures 3c,d). Compared with the IVL, the IVLSN had lower inflammation scores but not LIS_total_, whereas LIS items and LIS_total_ in both IVL and IVLSN groups were markedly lower than that in the corresponding groups of phase I (Supplementary Figures 4a,b).

#### Ultrastructural morphology of the lungs

**FIGURE 5:**
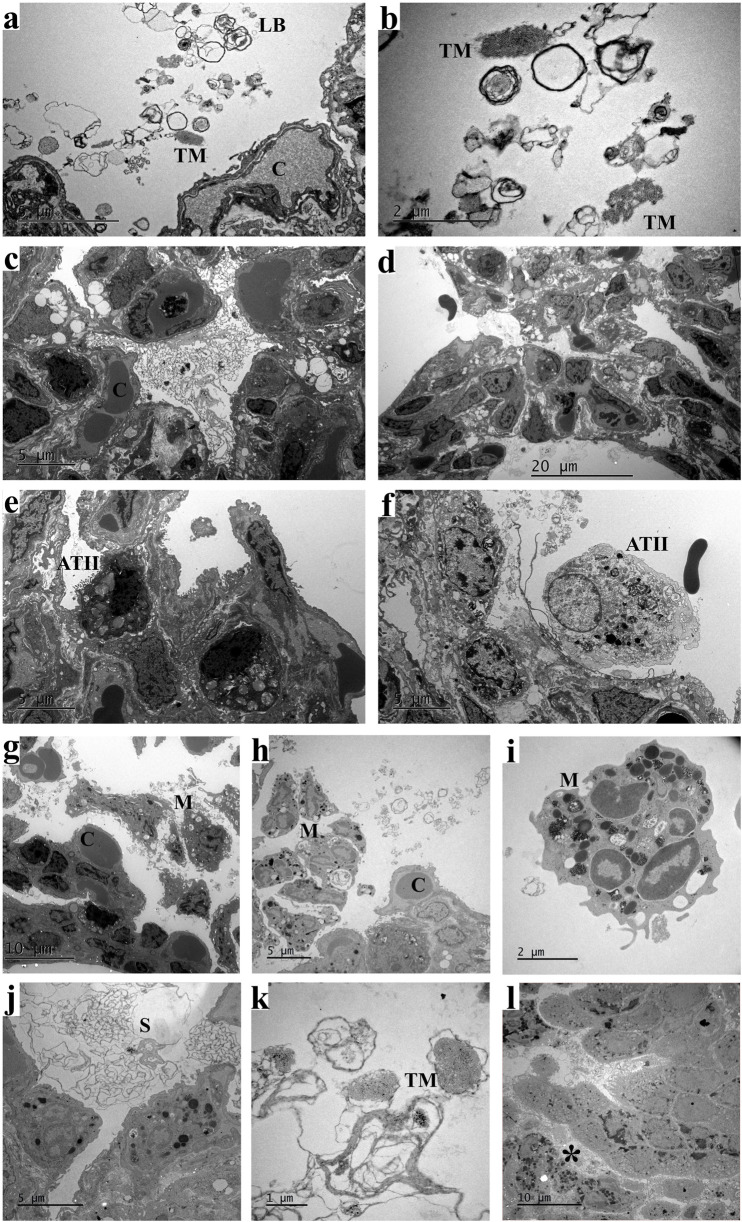
Ultrastructure of alveoli in 7-day old rabbit lungs. **(a,b)** IVC, loosen lamellar body and tubular myelin in alveolar space; **(c)** ITL, fluid infiltration in alveolar space and loosen ATII; **(d–f)** IVL, interstitial edema and loosen ATII with few lamellar bodies but intensified lysosome; **(g–i)** ITLSN, alveolar microphages; **(j,k)** ITLSN, surfactant phospholipid layers and tubular myelin in alveolar space; **(l)** ITLSN, interstitial inflammatory cells. For group definitions see Figure 1 legends. Scale bar size: **d** = 20 μm; **g** and **l** = 10 μm; **a**,**c**, **e**,**f**,**h** and **j** = 5 μm; **b** and **i** = 2 μm; **k** = 1 μm. Abbreviations: ATII, type II alveolar epithelial cell; C, capillary; LB, lamellar body; M, macrophage; S, surfactant phospholipid membrane layers; TM, tubular myelin. *, inflammatory cell. For group definitions see Figure 1 legends.

Similar features, but less in severity, of the microstructures of alveoli and bronchioles were seen compared to that in the phase I under the transmission electron microscopy. Ultrastructural morphology of the IVL group revealed interstitial edema, detached and loosely arranged ATII cells sloughing into the alveolar lumen, and markedly increased lysosomes (Figures 5d–f). Ultrastructural morphology of the IVLSN group (not shown) mirrored the ITLSN picture: surfactant-phospholipid lamellae and tubular myelin occupied the alveolar spaces, while interstitial infiltrates of inflammatory cells were again conspicuous.

#### Biochemical analysis of phospholipids and proteins in BALF and LH

The levels of TPL and DSPC in BALF were more than 2 folds, and TP and DSPC/TP more than 50% and 100%, higher in the IVLSN than in the IVL group, respectively (IVL: TPL 8.79 ± 2.30, DSPC 4.26 ± 1.89, TP 6.15 ± 2.03, DSPC/TP 0.69 ± 0.21; IVLSN: TPL 32.2 ± 12.6, DSPC 15.1 ± 5.96, TP 10.5 ± 4.20 and DSPC/TP1.51 ± 0.51) (Figures 3e,f; Table 4). DSPC/TPL was around 40–50% with no significant difference among the four groups. In the total lung phospholipid pool, i.e., in BALF + lung tissue homogenate, of the IVC and IVL groups, TPL were around 130–160 mg/kg, DSPC 33–40 mg/kg, and DSPC/TPL 25–27%, in average. For TPL and DSPC in BALF + lung tissue homogenate in the IVLSN, there were 270 and 87 mg/kg, which were 9 and 6 times that in the BALF (32 and 15 mg/kg in average) (Figure 3e; Table 4), respectively, with 31% of DSPC/TPL. It also derives net increments by approximately 126 and 50 mg/kg, or 86% and 138%, respectively, over the average TPL and DSPC in the IVC and IVL. Likewise, there were ratios of 8 and 5 between LH and BALF (i.e. 240 vs. 32.2 and 71.5 vs. 15.1 mg/kg, Table 4) for TPL or DSPC, respectively, in the IVLSN, denoting relatively enriched DSPC in both BALF and LH, compared with that of the ITLSN.

#### Measurement of mRNA expression in lung tissues (and in both phases of experiments)

In both IVC and IVL groups, there were no significant differences in the mRNA expression of SP-A, SP-B, and SP-C. Compared with the IVC group, the mRNA expression of NF-κB, TNF-α, IL-1β, IL-6, and IL-8 were upregulated in the IVL, and even higher in the IVLSN. The mRNA expression of Tie-2, Ang-1 and Ang-2 of both ITC and IVC had modest increment compared with that of C0, and ITL and IVL had approximately 40% depression compare to the corresponding ITC and IVC. The ITLSN and IVLSN had even lower expression of Tie-2 and Ang-1, but markedly enhanced expression of Ang-2 by three folds in both ITLSN and IVLSN (Figures 2g–i, 3g–i).

#### Comparison of mRNA expression of pro-inflammatory cytokines in liver and kidney tissues

In general, compared to the IVC, the IVL had 1-10 folds increments in the mRNA expression of ILs in both liver and kidney tissues. In contrast, the IVLSN had even more folds of increments than that of the IVL (Figures 3j,k).

#### Comparison of ITL and IVL

In Supplementary Figure 5, a direct comparison between ITC and IVC, or ITL and IVL in the Figures 2, 3 and Tables 4, 5 is provided. It facilitates comprehension of baseline characteristics between any two of the four groups in the two phases of experiments excluding both ITLSN and IVLSN groups (see above “Measurement of mRNA expression in lung tissues”).

**TABLE 5:** Stratified analysis of early death and 10-h survivors on lung surfactant phospholipid pools.

Group	Survival h (n)	TPL_BALF_ mg/kg	DSPC_BALF_ mg/kg	TP_BALF_ mg/kg	TPL_LH_ mg/kg	DSPC_LH_ mg/kg
ITL	<10 (1)	3.68	1.15	7.41	125	42.8
ITL	10 (29)	7.36 ± 3.24	3.82 ± 2.09	9.36 ± 3.29	138 ± 46.1	31.5 ± 15.4
ITLSN	<10 (6)	21.0 ± 2.49^bb^	11.0 ± 2.44^b^	12.4 ± 1.93	242 ± 45.3	88.2 ± 38.3^bb^
ITLSN	10 (18)	15.0 ± 5.20	7.35 ± 4.00	11.2 ± 2.96	208 ± 66.4	53.3 ± 27.6
IVL	<10 (13)	8.79 ± 2.22	4.90 ± 2.00	6.43 ± 2.24	145 ± 48.0	32.2 ± 16.0
IVL	10 (17)	8.79 ± 2.43	3.77 ± 1.69	5.96 ± 1.91	156 ± 48.2	37.2 ± 15.8
IVLSN	<10 (18)	35.5 ± 11.4^dd^	16.3 ± 5.31^d^	9.99 ± 4.01	236 ± 69.9	75.5 ± 38.9^dd^
IVLSN	10 (6)	22.3 ± 11.6	11.4 ± 6.74	11.9 ± 4.82	250 ± 81.8	59.5 ± 26.5

Group	Survival h (n)	TPL_BALF+LH_ mg/kg	DSPC_BALF+LH_ mg/kg
ITL	<10 (1)	129	44.0
ITL	10 (29)	145 ± 45.8	35.3 ± 15.4
ITLSN	<10 (6)	263 ± 45.7	99.3 ± 40.5^bb^
ITLSN	10 (18)	223 ± 68.2	60.7 ± 30.5
IVL	<10 (13)	154 ± 48.6	37.1 ± 16.1
IVL	10 (17)	165 ± 48.6	41.0 ± 16.6
IVLSN	<10 (18)	272 ± 68.6	91.8 ± 39.4
IVLSN	10 (6)	273 ± 73.9	70.9 ± 24.1

Values are means ± standard deviation. ^b^ *P* < 0.05, ^bb^ *P* < 0.01 vs. ITLSN (10 h), ^d^ *P* < 0.05, ^dd^ *P* < 0.01 vs. IVLSN (10 h). For group definitions see Table 1 legends.

## Discussion

Despite decades of efforts to bridge the knowledge gap, effective respiratory therapies with MV and ancillary medications for rescue purpose in pARDS remain to be limited, with high mortality risks to be solved [28]. The strength of current LPS-ALI model in 7-day old rabbits at their early post-neonatal infancy is characterized as simulating the pARDS_p_ and pARDS_exp_ by LPS-route associated different lung damage patterns, as shown in the survival length, LIS and pulmonary and extrapulmonary organ impairment. In neonatal and pediatric critical care, as we chose this animal age in maturation, it often faces with severe pneumonia and sepsis as underlying morbidities, with lung functional failure in ALI, PHRF and septic shock as the most fatal situation. The pharmacotherapeutic response patterns observed herewith may not readily explain the complex pathophysiology of pARDS in humans. However, the outcome was verified by Cdyn, survival rate and ST_50_ (for survival length), LIS, and the surfactant phospholipid metabolic pools. The key variables as biomarkers strengthened understanding of the underlying morbidities and applicability of pharmacotherapeutic action by surfactant and iNO treatment. It is therefore of causal implication as highly clinical problems oriented, in quest of rescue solution for prediction of pARDS outcome, despite there was an ineffectiveness/failure with the combined surfactant and iNO treatment [17].

From the lung damage patterns shown in the two phases of experiments, the direct ALI was associated with severe pneumonia and higher LIS, but moderate death risks; whereas the indirect ALI was with moderate LIS but high death risks due to sepsis associated multiorgan impairment in the lung, liver and kidney. The combined surfactant and iNO failed to improve the survival, associated with highly provoked mRNA expression of proinflammatory mediators as multiorgan involved septic lung damage patterns, especially in the IVLSN. These suggest imbalanced efficacies of the two combined respiratory ancillary medications when the lung impairment severity, hypotension and septic shock compromised ventilation/perfusion matching as underlying pathophysiologic, causative risks.

In addition, improper and insufficient surfactant dosage, surface activity, composition of phospholipids and hydrophobic proteins, SP-B and SP-C, its anti-inhibition potential and metabolic characteristics, partially associated with manufacturing process, may also determine as covariate towards the worse outcome. These issues should be explored to enrich our knowledge based on the current concept and recommendation for pARDS [29, 30]. This LPS-ALI model should be eligible for investigation of any new invention of surfactant preparation in novel components, manufacturing process improvement, or treatment response which would be associated with timing and dosing (loading and repeat doses) and delivery techniques (bolus, diluted lavage, or both), and interaction with other medications in critical care, in combination with pathobiological variables as phenotypic and sub-phenotypic indicators [31].

We found those of the IVLSN group had the shortest average survival time but with higher TPL and DSPC total pools (BALF + lung tissue) (Tables 4, 5). These findings may also be corroborated by sub-group data analysis for that of the 10-h survivors (Tables 4, 5). It is thereon estimated, approximately 63% and 36% of full dose surfactant TPL in the IVLSN and ITLSN groups, respectively, remaining in the whole lung metabolic pool at the end of MV, over the ensuing 7 h MV, or shorter for those of early death, and that the TPL being supposedly catabolized at a rate of 5%/h or 9%/h in average in the IVLSN or ITLSN lungs, respectively. Thus, these findings may be regarded as baseline evidences concerning the surfactant phospholipid metabolism in ALI for future investigation with stable isotope labeled phospholipids to estimate pharmacokinetic and pharmacodynamic efficiency as well. The presence of supposedly exogenous surfactant phospholipid membrane layers with no overt TM in adjacent alveolar space were in contrast to that of the controls (Figure 5). Given that the lung injury patterns (Figures 2–4), and phospholipid pools in those of early deaths and 10-h survivors (Tables 4, 5), the exogenous surfactant should be distributed in the injured lung alveoli, and taken up by ATII in reference to the control group (ITC, IVC). Whether it follows the re-synthesis and secretion by ATII, as reutilization, in the severely injured lungs is questionable and needs deliberate design of experiment to verify.

The histopathological features of lung impairment in ITL and ITLSN groups attained wide spread purulent and necrotic alveolitis and bronchiolitis, in contrast to mild and moderate inflammation in those of the IVL and IVLSN. However, there were disparities in the death risks, with mild-to-moderate deterioration in the lung mechanics measured by Cdyn in both ITL and IVL, but improved only in the IVLSN group but with higher early deaths. These discrepancies may account for the surfactant and iNO treatment ineffectiveness/failure, and should be of clinical implication in using MV-centered approaches to predict risks in immediate progression [12]. Another potentially eligible approach is to use surfactant lavage and subsequently with the bolus surfactant regimen, as we did in a meconium-induced lung injury model of near-term newborn rabbit model with good efficacy [16]. Repeated dosing is a third option, however, it would incur a decrease of already impaired functional residual lung volume, simply due to the presence of lung edema and consolidation. This was evidenced by higher LIS for alveolar injury and inflammation, and TP in BALF of both ITLSN and IVLSN groups, though their DSPC/TP levels were also elevated. As these animals had metabolic acidosis with low pH and very high lactate in the blood at the end of MV, their early death might be related to impaired hypoxemia-associated hemodynamic condition when intravenous volume expander and vasopressor were not applicable due to the small animal body size restriction.

This LPS-ALI model rendered a suitable approach in search of relevant pharmacotherapeutic products alleviating the severity of pARDS in neonates and infants. The treatment response pattern by the combined surfactant and iNO treatment, to the two different routes of LPS administration, was not expected in the original design. However, the resulting therapeutic response patterns with both physiological and pathobiological variables, underscored the disparities of outcome. Notably, by sub-group analysis, it revealed that those in the ITLSN and IVLSN with short survival length had nearly normal PCO_2_ but lower pH and very high lactate in blood at the end of MV (Table 3). Nevertheless, those in the ITLSN, IVL and IVLSN subgroups with short survival length had variable total surfactant phospholipid pools (Table 5).

To our surprise, under the high dose of LPS exposure, there were limited elevation in the mRNA expression of pro-inflammatory cytokines and mediators. In general, being a hallmark feature of septic ALI (ARDS_exp_), alveolar macrophages, as an innate immune cell type, are engaged in the maintenance of pulmonary homeostasis of immune response [32]. The classic NF-κB pathway activated pro-inflammatory cytokines, such as TNF-α, IL-1β, IL-6, IL-8, inducing neutrophil chemotaxis and activation in alveolar space, ultimately leading to the necrosis and purulent damages of alveolar structure, causing ALI and ARDS [33]. The evolving permeability as main pathological change in the course of lung damage may be centered in the pulmonary vascular endothelia, in which IL-1β, −6 and −8 play important roles through a number of vascular endothelial cell associated mediators [34]. This may be the next target to investigate for understanding the injury severity and protective ventilation strategy.

The unexpected high early death due to suspected hemodynamic derangement in the IVLSN was based solely on the evidence of enhanced mRNA expression in the liver and kidney as extrapulmonary organ injury, to link that in the clinical findings [35, 36]. In the study design, there was no measurement of extrapulmonary organ system for multiorgan dysfunction/failure. Therefore, current study was an attempt of reverse translation to explain mechanisms behind the clinical trial limitation, or failure, specifically for surfactant and/or iNO in pARDS [15, 37]. The biomarkers for defining the lung injury and alleviation by intervention, such as Ang-1, Ang-2, Tie-2 for vascular endothelial cell injury, and those for lung fluid clearance, surfactant production and metabolism, integrity and proliferation of ATs may be extended to understanding the pathogenesis and mechanistic roles underlying the pathophysiology and pharmacotherapeutic action [38].

Nitric oxide (NO) plays a pivotal role in modulating both intrapulmonary vascular tone and inflammatory responses. The concrete evidence of iNO in alleviating the lung parenchymal injury in ARDS is not clear yet [39, 40], and its off-label use in very preterm infants at risk of early bronchopulmonary dysplasia (BPD), tended to be safe and assumed to be active in modulation of inflammation and coagulation [41, 42]. Moreover, our own experience in experimental bacterial ALI and that of clinical report, suggest safety and optimal to the effectiveness of iNO with 30–50% oxygen [43] which may mitigate hyperoxic hazard. Like the off-label use in surfactant, the ancillary pharmacotherapeutic strategies in pARDS deserve their systematic investigation to reach consensus agreement. We thereby speculate it be different from the conventions of surfactant therapy in neonatal RDS of prematurity.

The most prominent limitation was the lack of direct evidence regarding the SN failure in both ITLSN and IVLSN groups. We consider the following indirect evidence to account for the SN failure, especially in IVLSN. First, the LIS_total_ values in IVL and IVLSN groups were lower than that of ITL and ITLSN, coinciding with that of Figure 4 at 2 and 10 h. It implied that the lung infection and inflammation as well as the treatment response patterns were not the same due to LPS route, representing a timely cause-effect from the commence of SN treatment to the end of ventilation between the two group. These were in contrary to the respective survival curves as shown in Figures 2a, 3a. We therefore infer that the survival discrepancy between these two groups, as were for ITLSN vs. ITL or IVLSN vs. IVL, should be impacted by factors other than the lung injury severity, very probably associated with systemic hypotension and septic shock in the treatment of SN. Although we implemented intermittent ECG monitoring, there had no intravenous infusion, vasopressor use, and blood pressure monitoring, due to tiny vasculature of the animals. Second, through examination of qPCR results, marked over-expression of mRNA of ANG-2, IL-1, -6 and 8 in the lungs, liver and kidney in IVLSN, implying multiple organ dysfunction. These phenomena were modest or moderate in ITLSN group. Third, by comparing the ultrastructure images, and the surfactant phospholipid pools in BALF + LH, of injured lungs in Figures 2, 3, the concurrent composite images in Figure 5 mainly represent lung maturation (as day 7 of postnatal life) with lamellar bodies and tubular myelin in alveolar space of unaffected lungs (ITC, IVC). In LPS group, the alveolar structure with inflammatory epithelial and interstitial cells, alveolar macrophages, often with prominent intracellular lysosomes, showing infection in the LPS-injured lungs. In ITLSN and IVLSN group, the large ring-like phospholipids in alveolar space as considered exogenous surfactant, with the ring diameter much exceeding that of the alveolar epithelial cells, were not found in the control animal lungs (ITC, IVC). Moreover, Figures 4, 5 revealed that the alveolar space and bronchiolar lumen in the LPS group were often filled with protein-rich exudate. This protein-and-cell debris was likely to sequester or inactivate endogenous and exogenous surfactant, thereby jeopardize the latter therapeutic action. In our previous preterm and near-term rabbit study, a porcine lung natural surfactant isolated SP-A strengthened biophysical and physiological properties of a porcine lung derived surfactant phospholipid preparation (Curosurf®, with SP-B and C), by reversing inactivation and inhibition from blood plasma proteins (fibrinogen/fibrin, albumin) and meconium [44]. Thus, this study did not have sufficient evidence to explain the mechanisms of exogenous surfactant therapeutic function in counterbalance inactivation/dysfunction, and iNO safety inferential in hemodynamic homeostasis. It remains a crucial and imperative knowledge gap in comprehension of LPS-ALI injury model simulating neonatal and pediatric critical care.

In addition to above strengths and limitations, there are other limitations to address. For the three different LPS dosing groups in the initial experiment, we did not have discerned the LPS dose dependent lung injury by severity and survival status, but found LPS route-dependent effects in survival. Neither did we found any marked benefits of combined PS and iNO in both ITLSN and IVLSN, with IVLSN the worst in survival, though surfactant phospholipid pool and Cdyn seem to be potentially optimal in the 10-h survivors. It was quite different from our previous experiments in adult or young animals at the expectation to find therapeutic efficacy by MV and ancillary medications with PS and iNO [24, 45]. Another weak point is the lack of intravenous fluid maintenance as the fragile vasculature for indwelling intubation. The intraperitoneal injection followed our previous experience with ventilated near-term rabbits that may in part counter balance acidosis and basal metabolism requirement [16, 20, 46]. In future study, these problems may be dealt with deliberate fluid injection and altering of PS dosage, along with other medications, such as intratracheal glucocorticosteroids, or vasopressors, to maintain a longer survival. The route and dose of LPS may still be an important issue for pharmacotherapeutic efficacy in parallel comparison of ARDS_p_ and ARDS_exp_. This may need a framework to standardize the LPS, or other inducing chemicals, with dosing regimen, more measurement of multiorgan functioning variables including sensitive biomarkers to define. Besides, the high LPS doses used in this study may induce a hyper-acute, fulminant lung injury that does not fully recapitulate the subacute phase of human pARDS. Therefore, the model may be best suited for studying early ALI, rather than its chronic or reparative phases.

## Conclusion

This study successfully established pulmonary and extrapulmonary ARDS models induced by a high dose range of LPS, in 7-day-old rabbits, explicitly depicting the distinct pathophysiological mechanisms underlying the pharmacotherapeutic action using the combined surfactant and iNO. The impact was with the disparities of LPS route dependent lung injury and survival patterns, underscoring the pathobiological phenotypes of associated pulmonary impairment and inflammatory responses versus the extrapulmonary multiorgan involvement. The model and concept deserve further validation in the overall and specific efficacy assessment for rescue of pARDS_p_ and pARDS_exp_.

## Data Availability

The original contributions presented in the study can be found via NutStore link: Dr. Zhuang G data files https://www.jianguoyun.com/p/DeoOWs0Q6pX-DRia56EGIAA (accession number: YQMQeY) PWD: YQMQeY. Further inquiries can be directed to the corresponding authors.
