# Supplementary material for: Intra- and extrapulmonary lipopolysaccharides-induced acute lung injury and pharmacotherapeutic response patterns in ventilated 7-day-old rabbits
Source: Exp Biol Med (Maywood). 2026 Feb 24;251:10788. doi: 10.3389/ebm.2026.10788 (PMC12971534; doi:10.3389/ebm.2026.10788)
Supplement: Supplementary file 1 [file Supplementaryfile1.docx]

**Supplementary information**

The data had uploaded to this website: **https://www.jianguoyun.com/p/DeoOWs0Q6pX-DRia56EGIAA (accession number：YQMQeY)**

eFig. 1 The electrocardiograph of normal animals.


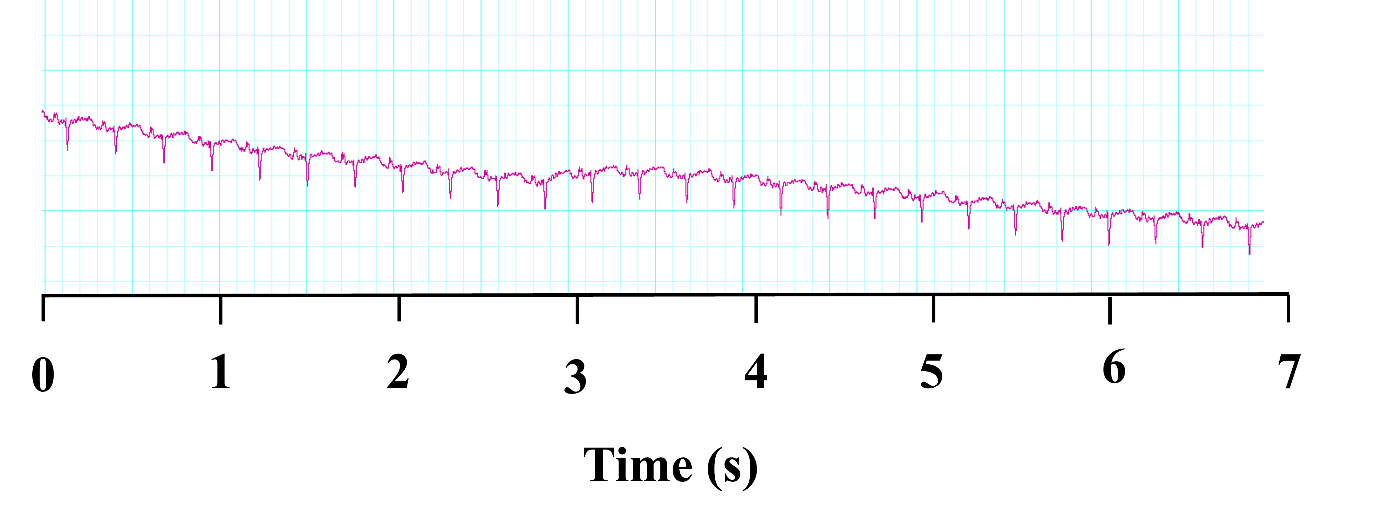


**eFig. 2 Comparison of the major findings in Phase I experiment.**

**a.** Trend of Cdyn (ITC n=12, ITL (15/20/25) n=17 for each dose of LPS, ITLSN (15/20/25) n=8 for each dose of LPS); **b.** Vv and CV(Vv) (C0 n=8, ITC n=8, ITL (15/20/25) n=10 for each dose of LPS, ITLSN (15/20/25) n=8 for each dose of LPS); **c.** LIS (C0 n=8, ITC n=8, ITL (15/20/25) n=10 for each dose of LPS, ITLSN(15/20/25) n=8 for each dose of LPS); **d.** TPL, DSPC and TP in BALF (C0 n=8, ITC n=8, ITL (15/20/25) n=10 for each dose of LPS, ITLSN (15/20/25) n=8 for each dose of LPS);**e.** mRNA expression of pro-inflammatory mediators in lung tissue (NF-κB, TNF-α, IL) (C0 n=8, ITC n=8, ITL (15/20/25) n=10 for each dose of LPS, ITLSN (15/20/25) n=8 for each dose of LPS); For group definitions see Fig. 1-3 legends, and for abbreviations see Fig 2-3 legends. Values are mean + standard deviation. There were no statistic significances between the groups.


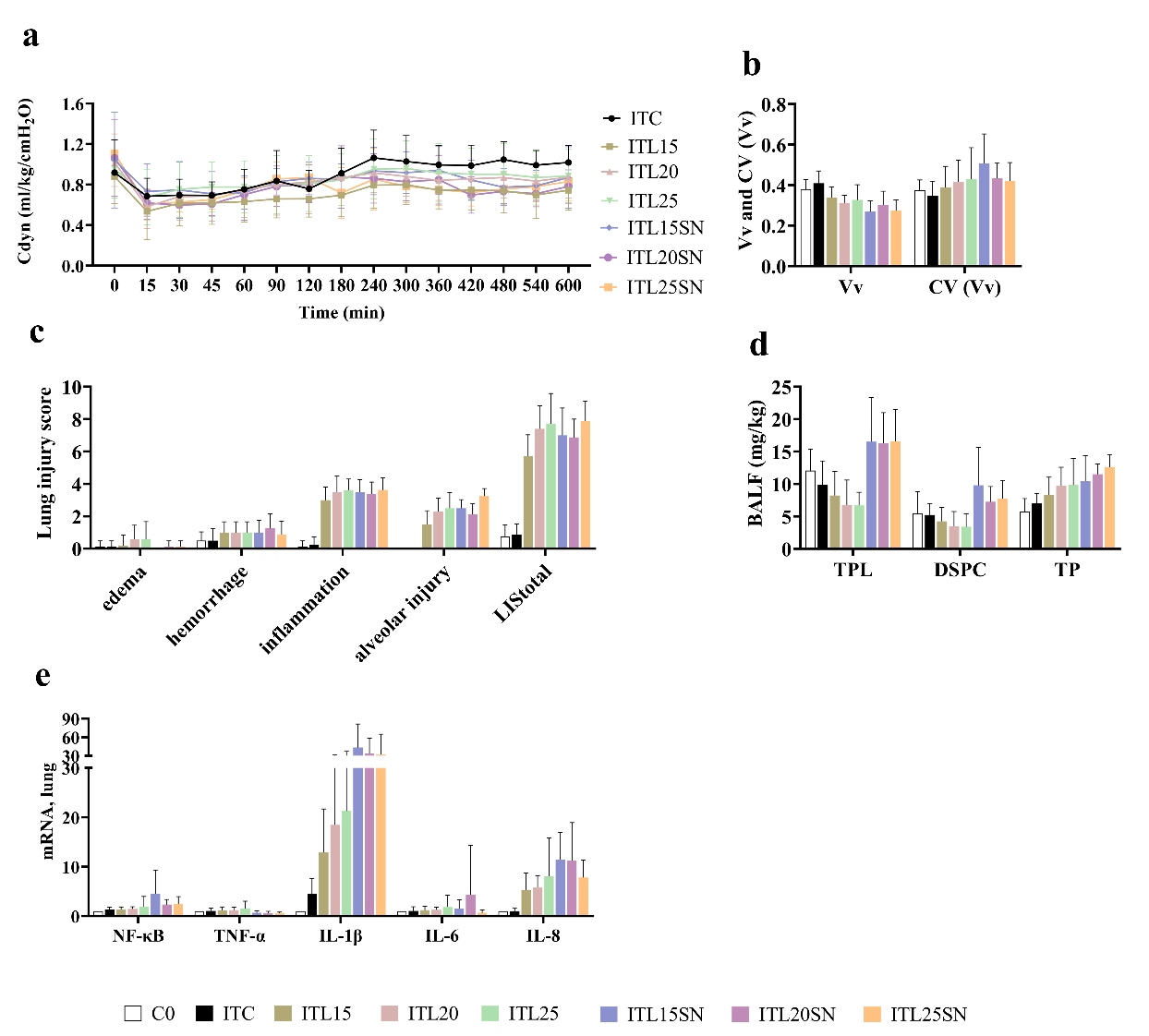


**eFig. 3 Comparison of the major findings in Phase II experiment.**

**a.** Trend of Cdyn (IVC n=12, IVL (15/20/25) n=17 for each dose of LPS, IVLSN (15/20/25) n=8 for each dose of LPS); **b.** Vv and CV(Vv) (C0 n=8, IVC n=8, IVL (15/20/25) n=10 for each dose of LPS, IVLSN (15/20/25) n=8 for each dose of LPS); **c.** LIS (C0 n=8, IVC n=8, IVL (15/20/25) n=10 for each dose of LPS, IVLSN (15/20/25)n=8 for each dose of LPS); **d.** TPL, DSPC and TP in BALF(C0 n=8, IVC n=8, IVL (15/20/25) n=10 for each dose of LPS, IVLSN (15/20/25) n=8 for each dose of LPS);**e.** mRNA expression of pro-inflammatory mediators in lung tissue (NF-κB, TNF-α, IL) (C0 n=8, IVC n=8, IVL(15/20/25) n=10 for each dose of LPS, IVLSN (15/20/25) n=8 for each dose of LPS); For group definitions see Fig. 1-3 legends, and for abbreviations see Fig 2-3 legends. Values are mean + standard deviation. ^a^*P* < 0.05 *vs.* IVL15.

**
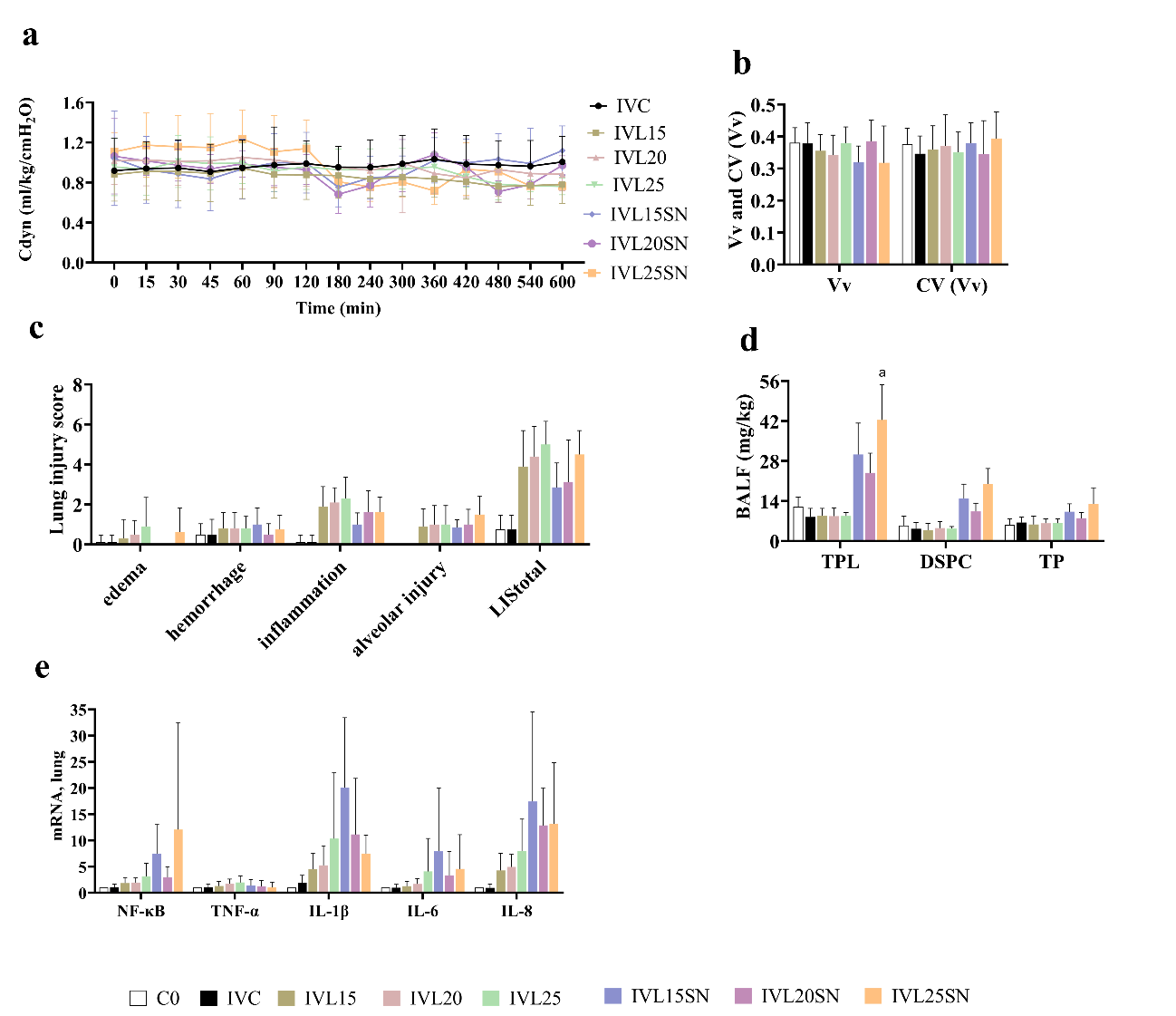
**

**eFig. 4 Comparison of Vv & CV (Vv) & LIS among ITL & ITLSN & IVL & IVLSN groups.**

For group definitions see Fig. 1-3 legends, and for abbreviations see Fig 2-3 legends. Values are mean + standard deviation.


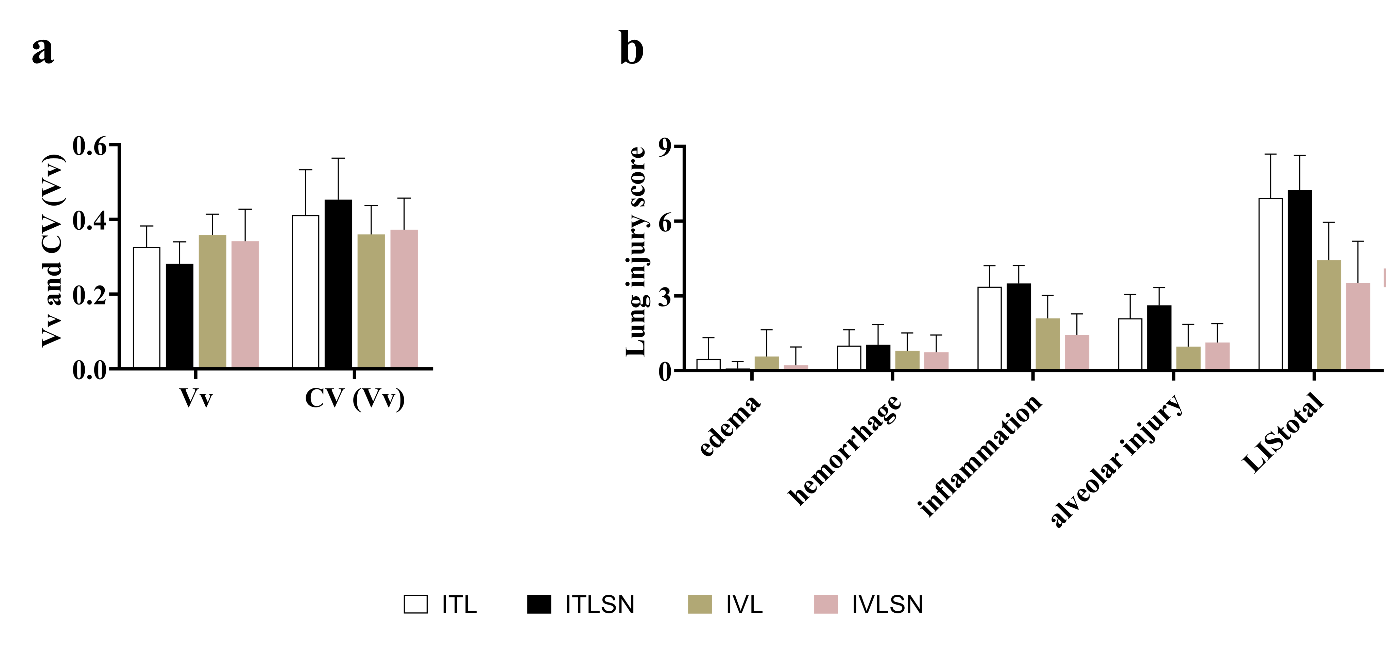


**eFig. 5 Comparison of the major findings between ITL & IVL groups.**

**a.** Kaplan-Meier survival curves (Note: the survival time of IVC was shown by 10-11 hours for easy recognition); **b.** trend of Cdyn; **c.** Vv and CV(Vv); **d.** LIS; **e.** TPL, DSPC and TP in BALF; **f.** DSPC/TP ratio; **g.** mRNA expression of SPs in lung tissue; **h.** mRNA expression of pro-inflammatory mediators in lung tissue (NF-κB, TNF-α, IL); **i.** mRNA expression of the injury biomarkers of endothelial cells in lung tissue (Tie-2, Ang); **j.** mRNA expression of pro-inflammatory mediators in liver tissue; **k.** mRNA expression of pro-inflammatory mediators in kidney tissue. For group definitions see Fig. 1-3 legends, and for abbreviations see Fig 2-3 legends. Values are mean + standard deviation. ^#^*P* < 0.05, ^##^*P* < 0.01, ^###^*P* < 0.001 *vs.* ITL.


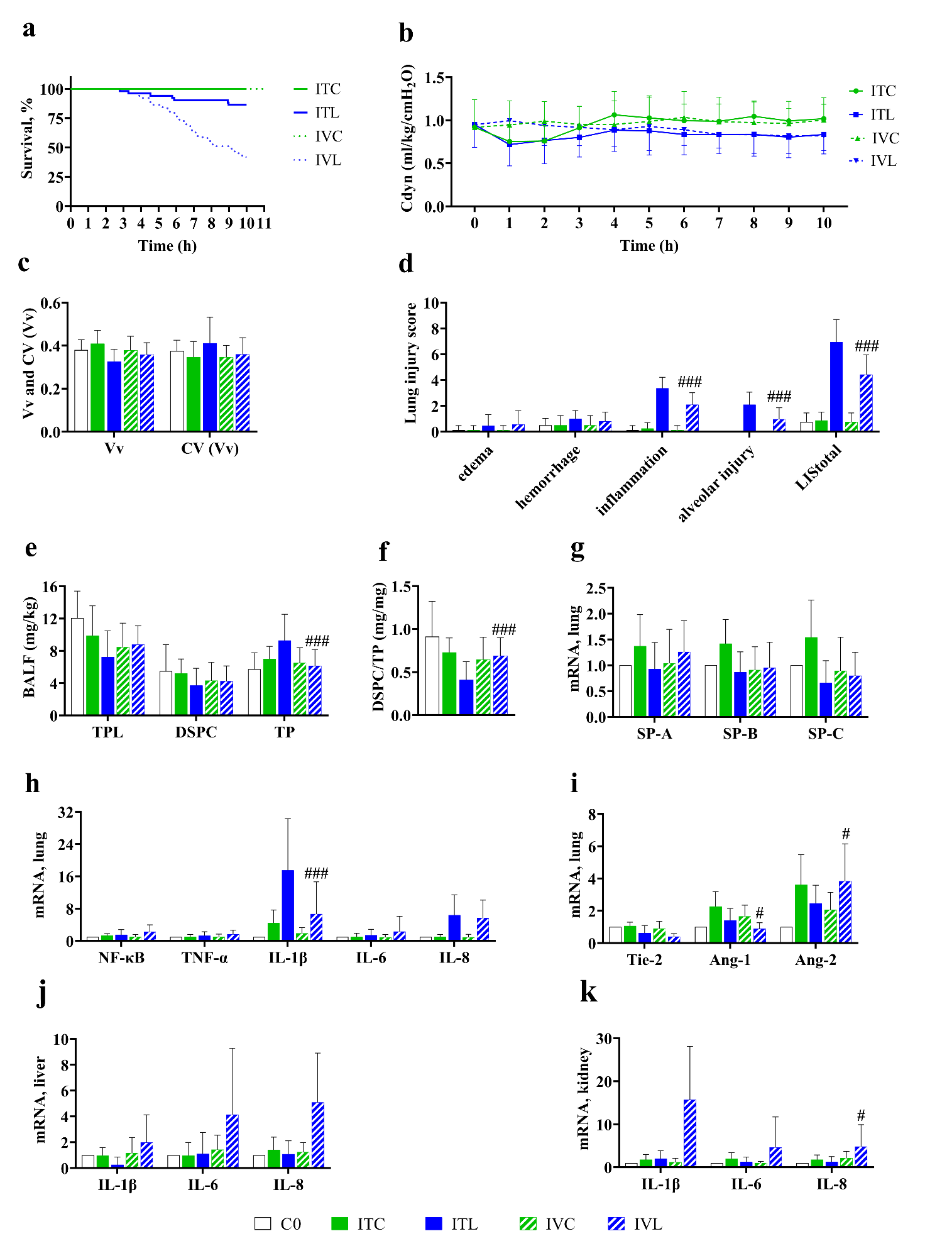


**eTable 1. Primer DNA sequences (5’-3’) of target genes (F: forward, R: reverse).**

**__________________________________________________________________________________**

Target gene F/R primer Primer sequences

__________________________________________________________________________________

β-actin F ATTGGCATGGCTTTATTCGTG

R GTCACCTTCACCGTTCCAGTTT

SP-A F ATGACTCAAGCGTAATAAATCGTG

R CTCTGGACAAAGCGAGCACA

SP-B F GCTTTACCGCTACTTGCTCACA

R CTTGGACCATCTTCTGTTGCTTT

SP-C F TGGTCCTCGTGGTCGTGGTG

R GCTGGTAGTCGCAGGTGACAATG

NF-κB F GATGTGAAGATGCTCCTGGCTGTC

R CGGTGGATGATTGCTAGGTGTAAGAC

TNF-α F GGCATGAAGCTCACGGACAACC

R GCCTTGACCGCTGAAGAGAACC

IL-1β F CAGGCTCCAGGATGCACAACAG

R CACGCAGGACAGGTACAGATTCTTC

IL-6 F GAAGAAGCCACCCTCAAGCC

R CCATGAAATTCCGCAAGCAA

IL-8 F TTGGTCAGGCCATGAGTTCC

R TGTGCCCTCACAACATCCCT

Tie-2 F CACTCCCAGAATCTCAAGCACCAG

R GCACTGTCAGGCAGGTCATTCC

Ang-1 F CCTGCCTCGCTGCCATTGTG

R CAGTTGCCGTCCTGTTCTGGAAG

Ang-2 F CCAGTGGCATCTACACGCTAACG

R CCATCTTCCCGCCGCTGAATG

__________________________________________________________________________________

Abbreviations: SP, surfactant proteins; NF-κB, nuclear transcription factor-κB; TNF-α, tumor necrosis factor-α; IL, interleukin; Tie, tyrosine kinase receptor; Ang, angiopoietin.

**eTable 2.** **Basic information of each individual LPS dose** **of experimental animals.**

__________________________________________________________________________________Group N Weight Male Survival pH PCO_2_ Lactate (g) (%) (%) (mmHg) (mmol/L)

__________________________________________________________________________________ITL15 17 115 + 6.5 6 (35.3) 14 (82.4) 7.25 + 0.18 61.4 + 19.4 5.8 + 6.6

ITL20 17 113 + 5.3 7 (41.2) 15 (88.2) 7.22 + 0.21 68.5 + 20.2 4.1 + 4.0

ITL25 17 113 + 6.2 9 (52.9) 15 (88.2) 7.28 + 0.16 62.3 + 14.1 3.4 + 3.7

ITL15SN 8 106 + 2.5 0 (0.0) 6 (75.0) 7.33 + 0.06 49.1 + 20.7 7.3 + 5.3

ITL20SN 8 112 + 5.2 1 (12.5) 5 (62.5) 7.20 + 0.21 55.3 + 23.5 9.4 + 8.6

ITL25SN 8 107 + 3.1 4 (50.0) 7 (87.5) 7.30 + 0.09 66.1 + 11.5 4.0 + 3.6

IVL15 17 114 + 4.5 9 (52.9) 9 (60.0) 7.19 + 0.17 48.5 + 25.2 12.2 + 7.0

IVL20 17 117 + 6.4 9 (52.9) 8 (47.1) 7.17 + 0.15 47.1 + 20.1 11.1 + 5.8

IVL25 17 117 + 5.3 7 (41.2) 4 (23.5) 7.21 + 0.14 34.8 + 17.8 14.1 + 4.5

IVL15SN 8 108 + 4.2 1 (12.5) 3 (37.5) 7.14 + 0.23 42.0 + 18.1 11.4 + 5.5

IVL20SN 8 110 + 3.7 3 (37.5) 2 (25.0) 7.17 + 0.18 34.8 + 22.6 16.1 + 6.3

IVL25SN 8 106 + 6.8 2 (25.0) 1 (12.5) 7.29 + 0.17 34.4 + 24.2 11.5 + 5.6

__________________________________________________________________________________

Group definitions and abbreviations: IT, intratracheal; IV, intravenous; C, control; L, lipopolysaccharides (15, 20, 25 mg/kg); S, pulmonary surfactant (200 mg/kg); N, inhaled nitric oxide (10 ppm); C0, non-ventilated control. Values are means + standard deviation

**eTable 3. Partial original data for** **each individual LPS dose of experimental animals.**

__________________________________________________________________________________

Group N Vv CV (Vv) edema hemorrhage inflammation

__________________________________________________________________________________

C0 8 0.38 + 0.05 0.38 + 0.05 0.13 + 0.35 0.50 + 0.54 0.13 + 0.35

ITC 8 0.41 + 0.06 0.35 + 0.07 0.13 + 0.35 0.50 + 0.76 0.25 + 0.46

ITL15 10 0.34 + 0.05 0.39 + 0.10 0.20 + 0.63 1.00 + 0.67 3.00 + 0.82

ITL20 10 0.31 + 0.04 0.42 + 0.11 0.60 + 0.84 1.00 + 0.67 3.50 + 0.97

ITL25 10 0.33 + 0.08 0.43 + 0.15 0.60 + 1.07 1.00 + 0.67 3.60 + 0.70

ITL15SN 8 0.27 + 0.05~ 0.51 + 0.15 0.00 + 0.00 1.00 + 0.76 3.50 + 0.76

ITL20SN 8 0.30 + 0.07 0.43 + 0.08 0.13 + 0.35 1.25 + 0.89 3.38 + 0.74

ITL25SN 8 0.27 + 0.05 0.42 + 0.09 0.13 + 0.35 0.88 + 0.83 3.63 + 0.74

IVC 8 0.38 + 0.07 0.35 + 0.06 0.13 + 0.35 0.50 + 0.76 0.13 + 0.35

IVL15 10 0.36 + 0.05 0.36 + 0.07 0.30 + 0.95 0.80 + 0.79 1.90 + 0.99

IVL20 10 0.34 + 0.06 0.37 + 0.10 0.50 + 0.71 0.80 + 0.79 2.10 + 0.74

IVL25 10 0.38 + 0.05 0.35 + 0.06 0.90 + 1.45 0.80 + 0.63 2.30 + 1.06

IVL15SN 7 0.32 + 0.05 0.38 + 0.06 0.00 + 0.00 1.00 + 0.82 1.00 + 0.58

IVL20SN 8 0.39 + 0.07 0.35 + 0.10 0.00 + 0.00 0.50 + 0.53 1.63 + 1.06

IVL25SN 8 0.32 + 0.12 0.39 + 0.08 0.63 + 1.19 0.75 + 0.71 1.63 + 0.74

__________________________________________________________________________________

__________________________________________________________________________________

(Continued) alveolar injury LIS_total_ TPL_BALF_ DSPC_BALF_ TP_BALF_

(mg/kg) (mg/kg) (mg/kg)

__________________________________________________________________________________

C0 0.00 + 0.00 0.75 + 0.71 12.1 + 3.34 5.50 + 3.30 5.74 + 2.03

ITC 0.00 + 0.00 0.88 + 0.64 9.89 + 3.69 5.23 + 1.17 7.00 + 1.56

ITL15 1.50 + 0.85 5.70 + 1.34 8.24 + 3.70 4.26 + 2.17 8.33 + 2.72

ITL20 2.30 + 0.82 7.40 + 1.43 6.76 + 3.84 3.52 + 2.25 9.71 + 2.88

ITL25 2.50 + 0.97 7.70 + 1.89 6.72 + 2.02 3.41 + 2.03 9.85 + 4.09

ITL15SN 2.50 + 0.54 7.00 + 1.69 16.5 + 6.84 9.79 + 5.87 10.4 + 3.98

ITL20SN 2.13 + 0.64 6.88 + 1.13 16.3 + 4.73 7.26 + 2.35 11.5 + 1.57

ITL25SN 3.25 + 0.46 7.88 + 1.25 16.6 + 4.94 7.77 + 2.80 12.6 + 1.99

IVC 0.00 + 0.00 0.75 + 0.71 8.48 + 2.97 4.32 + 2.24 6.51 + 1.89

IVL15 0.90 + 0.88 3.90 + 1.79 8.83 + 2.61 3.83 + 2.35 5.88 + 2.88

IVL20 1.00 + 0.94 4.40 + 1.51 8.74 + 2.96 4.61 + 2.24 6.30 + 1.51

IVL25 1.00 + 0.94 5.00 + 1.15 8.81 + 1.22 4.34 + 0.78 6.30 + 1.53

IVL15SN 0.86 + 0.38 2.50 + 1.51 30.3 + 10.95 14.9 + 5.08 10.3 + 2.74

IVL20SN 1.00 + 0.76 3.13 + 2.10 23.~~9~~ + 6.94 10.4 + 2.90 8.03 + 2.04

IVL25SN 1.50 + 0.93 4.50 + 1.20 42.4 + 12.3^a^ 20.0 + 5.41 13.0 + 5.65

__________________________________________________________________________________

__________________________________________________________________________________

(Continued) NF-κB TNF-α IL-1β IL-6 IL-8

__________________________________________________________________________________

C0 1.00 + 0.00 1.00 + 0.00 1.00 + 0.00 1.00 + 0.00 1.00 + 0.00

ITC 1.36 + 0.46 1.06 + 0.76 4.47 + 3.16 1.05 + 0.86 1.02 + 0.56

ITL15 1.33 + 0.53 1.18 + 0.68 12.9 + 8.83 1.21 + 0.74 5.32 + 3.40

ITL20 1.47 + 0.44 1.21 + 0.64 18.5 + 12.6 1.27 + 0.56 5.79 + 2.44

ITL25 1.92 + 2.06 1.56 + 1.46 21.3 + 15.8 1.85 + 2.36 8.12 + 7.67

ITL15SN 4.52 + 4.82 0.68 + 0.40 43.4 + 38.0 1.52 + 1.77 11.5 + 5.50

ITL20SN 2.34 + 1.05 0.59 + 0.40 34.1 + 24.6 4.35 + 9.97 11.3 + 7.66

ITL25SN 2.51 + 1.44 0.56 + 0.24 32.0 + 32.9 0.68 + 0.58 7.81 + 3.55

IVC 1.04 + 0.61 1.05 + 0.63 1.89 + 1.43 0.96 + 0.65 0.94 + 0.72

IVL15 1.85 + 1.00 1.30 + 0.87 4.51 + 3.06 1.27 + 0.91 4.27 + 3.28

IVL20 1.96 + 0.87 1.73 + 0.89 5.25 + 3.70 1.69 + 1.04 4.95 + 2.39

IVL25 3.12 + 2.54 1.99 + 1.24 10.3 + 12.6 4.08 + 6.30 7.97 + 6.08

IVL15SN 7.48 + 5.56 1.35 + 1.19 20.1 + 13.3 7.96 + 12.0 17.4 + 17.1

IVL20SN 2.92 + 2.00 1.24 + 1.15 11.1 + 10.7 3.31 + 4.61 12.9 + 7.2

IVL25SN 12.1 + 20.4 1.01 + 1.05 7.48 + 3.49 4.55 + 6.53 13.2 + 11.7

__________________________________________________________________________________

Group definitions and abbreviations: IT, intratracheal; IV, intravenous; C, control; L, lipopolysaccharides (15, 20, 25 mg/kg); S, pulmonary surfactant (200 mg/kg); N, inhaled nitric oxide (10 ppm); C0, non-ventilated control. Values are means + standard deviation. Vv, alveolar expansion. CV(Vv), variation of alveolar aeration. LIS, lung injury score. TPL, Total phospholipid. DSPC, Disaturated phosphatidylcholine. Tp, Total proteins. BALF, Bronchoalveolar lavage fluid. ^a^*P* < 0.05 *vs.* IVL15.

**eTable 4. Dynamic compliance of respiratory system and survival sample numbers (N) over the 10-h ventilation.**

__________________________________________________________________________________

Cdyn (ml/kg/cmH_2_O)

_____________________________________________________________________

0 min 15 min 30 min 45 min

__________________________________________________________________________________

ITC 0.92 + 0.32 0.69 + 0.18 0.70 + 0.16 0.69 + 0.13

(12) (12) (12) (12)

ITL 0.95 + 0.26 0.60 + 0.26 0.68 + 0.25 0.70 + 0.24

(51) (51) (51) (51)

ITLSN 1.07 + 0.35 0.65 + 0.20 0.66 + 0.19 0.66 + 0.15

(24) (24) (24) (24)

IVC 0.92 + 0.32 0.94 + 0.27 0.95 + 0.28 0.91 + 0.27

(12) (12) (12) (12)

IVL 0.95 + 0.26 0.96 + 0.26^###^ 0.98 + 0.26^###^ 0.97 + 0.26^###^

(51) (51) (51) (51)

IVLSN 1.07 + 0.35 1.04 + 0.29 1.01 + 0.29 0.97 + 0.30

(24) (24) (24) (24)

____________________________________________________________________________________________________________________________________________________________________ (Continued) Cdyn (ml/kg/cmH_2_O)

_____________________________________________________________________

1 h 1.5 h 2 h 3 h

__________________________________________________________________________________ITC 0.75 + 0.20 0.84 + 0.30 0.76 + 0.18 0.91 + 0.25

(12) (12) (12) (12)

ITL 0.72 + 0.25 0.75 + 0.27 0.77 + 0.27 0.80 + 0.23

(51) (51) (51) (50)

ITLSN 0.72 + 0.18 0.82 + 0.16 0.85 + 0.16 0.82 + 0.24

(24) (24) (24) (24)

IVC 0.95 + 0.28 0.98 + 0.38 0.99 + 0.23 0.95 + 0.21

(12) (12) (12) (12)

IVL 1.00 + 0.28^###^ 0.94 + 0.24^##^ 0.94 + 0.23^##^ 0.92 + 0.21

(51) (51) (51) (50)

IVLSN 1.05 + 0.28 1.02 + 0.28 1.02 + 0.26 0.75 + 0.18^^^^

(24) (24) (24) (21)

__________________________________________________________________________________

__________________________________________________________________________________

(Continued) Cdyn (ml/kg/cmH_2_O)

_______________________________________________________________

4 h 5 h 6 h 7 h

__________________________________________________________________________________

ITC 1.06 + 0.27 1.03 + 0.26 1.00 + 0.19 0.99 + 0.20

(12) (12) (12) (12)

ITL 0.89 + 0.25 0.88 + 0.23 0.84 + 0.23 0.84 + 0.22

(49) (49) (47) (47)

ITLSN 0.88 + 0.25 0.85 + 0.19 0.85 + 0.22 0.76 + 0.18

(24) (23) (23) (21)

IVC 0.95 + 0.27 0.99 + 0.29 1.03 + 0.30 0.98 + 0.28

(12) (12) (12) (12)

IVL 0.89 + 0.20 0.93 + 0.33 0.89 + 0.18 0.84 + 0.16

(48) (42) (39) (32)

IVLSN 0.79 + 0.19 0.87 + 0.20 0.97 + 0.23 0.97 + 0.18

(17) (14) (9) (9)

____________________________________________________________________________________________________________________________________________________________________ (Continued) Cdyn (ml/kg/cmH_2_O)

_______________________________________________________________

8 h 9 h 10 h

ITC 1.05 + 0.18 0.99 + 0.14 1.02 + 0.17

(12) (12) (12)

ITL 0.84 + 0.25^*^  0.80 + 0.24^*^ 0.84 + 0.23^*^

(47) (44) (44)

ITLSN 0.76 + 0.15 0.76 + 0.14 0.83 + 0.21

(19) (19) (17)

IVC 0.97 + 0.24 0.96 + 0.26 1.01 + 0.26

(12) (12) (12)

IVL 0.83 + 0.22 0.82 + 0.21 0.82 + 0.17^!^

(28) (24) (22)

IVLSN 0.92 + 0.24 0.88 + 0.25 1.01 + 0.21

(7) (6) (6)

__________________________________________________________________________________Group definitions and abbreviations: IT, intratracheal; IV, intravenous; C, control; L, lipopolysaccharides (mixed with15, 20, 25 mg/kg); S, surfactant (200 mg/kg); N, inhaled nitric oxide (10 ppm); C0, non-ventilated. Values are means + standard deviation.* *P*< 0.05 *vs.* ITC, ## *P* < 0.01, ### *P* < 0.001 *vs.* ITL, ! *P* < 0.05 *vs.* IVC, ^^^^*P* < 0.01 *vs.* IVL.
