# Supplementary material for: Intra- and extrapulmonary lipopolysaccharides-induced acute lung injury and pharmacotherapeutic response patterns in ventilated 7-day-old rabbits
Source: Exp Biol Med (Maywood). 2026 Feb 24;251:10788. doi: 10.3389/ebm.2026.10788 (PMC12971534; doi:10.3389/ebm.2026.10788)
Supplement: Supplementary file 2 [file DataSheet1.DOCX]

NutStore link: Dr. Zhuang G data files

https://www.jianguoyun.com/p/DeoOWs0Q6pX-DRia56EGIAA (accession number：YQMQeY) PWD: YQMQeY
